# Quercetin‐Loaded Bioglass Injectable Hydrogel Promotes m6A Alteration of Per1 to Alleviate Oxidative Stress for Periodontal Bone Defects

**DOI:** 10.1002/advs.202403412

**Published:** 2024-05-15

**Authors:** Huimin Zhu, Chao Cai, Yeke Yu, Yuning Zhou, Shiyuan Yang, Yue Hu, Yan Zhu, Jia Zhou, Jieyun Zhao, Hailong Ma, Yujie Chen, Yuanjin Xu

**Affiliations:** ^1^ Department of Oral Surgery Shanghai Ninth People's Hospital Shanghai Jiao Tong University School of Medicine College of Stomatology National Center for Stomatology National Clinical Research Center for Oral Diseases Shanghai Key Laboratory of Stomatology Shanghai Jiao Tong University No. 639 Zhizaoju Rd Shanghai 200011 China; ^2^ Zhejiang Engineering Research Center for Tissue Repair Materials Wenzhou Institute University of Chinese Academy of Sciences Wenzhou Zhejiang 325000 China; ^3^ Department of Oral Maxillofacial‑Head and Neck Oncology Shanghai Ninth People's Hospital College of Stomatology Shanghai Jiao Tong University School of Medicine No 639, Zhizaoju Rd Shanghai 200011 China; ^4^ State Key Laboratory of Metal Matrix Composites School of Materials Science and Engineering Shanghai Jiao Tong University Shanghai 200240 China

**Keywords:** bone defect repair, delivery system, orofacial bone mesenchymal stem cells, oxidative stress, quercetin

## Abstract

Periodontal disease ranks third among noncommunicable illnesses, behind cancer and cardiovascular disease, and is closely related to the occurrence and progression of various systemic diseases. However, elucidating the processes of periodontal disease and promoting periodontal bone regeneration remains a challenge. Here, quercetin is demonstrated to reduce the oxidative stress state of orofacial mesenchymal stem cells (OMSCs) in vitro and to affect the osteogenic growth of OMSCs through molecular mechanisms that mediate the m6A change in Per1. Nevertheless, the limited therapeutic efficacy of systemic medication and the limitations of local medication resulting from the small, moist, and highly dynamic periodontal environment make it challenging to treat periodontal tissues with medication. Herein, a biosafe injectable hydrogel drug‐controlled delivery system is constructed as a bone‐enhancing factory and loaded with quercetin to treat oxidative stress injury in periodontal tissues. This drug‐carrying system made up of nanoscale bioglass microspheres and a light‐cured injectable hydrogel, allows effective drug particle loading and cementation in the dynamic and moist periodontal environment. Furthermore, the system demonstrates the ability to stimulate OMSCs osteogenic differentiation in a Per1‐dependent manner, which ultimately promotes periodontal bone repair, suggesting that this system has potential for clinical periodontal therapy.

## Graphical abstract

Reducing oxidative stress and encouraging bone regeneration are two crucial therapeutic strategies in periodontitis. An injectable hydrogel loaded with traditional remedy quercetin works remarkably well to address these problems. Quercetin is crucial to this process as it regulates Per1 expression and its m6A alteration in OMSCs. The finding provides a potential application of quercetin for treating periodontal bone defects.

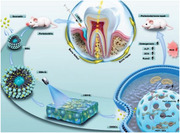

## 1 Introduction

Periodontal disease, a chronic, cumulative, progressive dental illness that directly and indirectly affects general health (in severe cases can lead to disability or even death), is an important contributing factor to many diseases with extremely high mortality rates.^[^ ^1^ ^]^ Less than 10% of individuals across all age categories had good periodontal health, according to the results of China's fourth oral health epidemiologic survey, which revealed an exceptionally high prevalence of periodontal disease. Clinical investigations have revealed oxidative damage to DNA, proteins, and lipids in the gingival tissues, saliva, and blood of patients with periodontitis, suggesting that oxidative stress is involved in the pathophysiological process of periodontitis. Oxidative stress increases protease cellular production, reactive oxygen species (ROS), neutrophil inflammatory infiltration, and ultimately alveolar bone loss. In this situation, using antioxidant compounds to regulate oxidative stress is a viable strategy to combat periodontitis.^[^ ^2^ ^]^

Among antioxidants, quercetin has an inherent reducing structure composed of hydroxyl groups, 2,3‐double bonds, and heterocycles of catechols, giving it significant antioxidant characteristics for efficient ROS scavenging.^[^ ^3^ ^]^ Researchers discovered in 2014 that quercetin lowered ROS levels and bacterial colonization in human gingival fibroblasts.^[^ ^4^ ^]^ The mechanism by which quercetin enhances bone defect repair has received much attention.^[^ ^5^ ^]^ Several studies have found quercetin to increase the expression of osteogenesis‐related genes, thereby boosting the differentiation and mineralization of bone marrow and adipose‐derived mesenchymal stem cells.^[^ ^6^ ^]^ However, they did not provide a detailed explanation of exactly how quercetin regulates the differentiation of stem cells. A recent study reported that the expression of m6A was significantly different in periodontitis treated with quercetin,^[^ ^7^ ^]^ indicating that quercetin may participate in the course of periodontitis via m6A modification. In the past decade, m6A‐mediated post‐transcriptional regulation and its significance in disease have been widely explored. It is a highly dynamic and reversible biological process subject to the control of “writers,” “readers,” and “erasers.”^[^ ^8^ ^]^ These m6A regulatory proteins control the destiny of m6A‐modified transcripts. Dysregulation of these proteins can lead to abnormal transcription, processing, and translation, which can impact the development, course, and prognosis of a variety of diseases, such as cancer, cardiovascular disease, and other conditions.^[^ ^9^ ^]^ Targeting m6A modifications may be an important and promising therapeutic modality.^[^ ^10^ ^]^ Nevertheless, no research has examined the underlying processes of m6A alteration in periodontal cells. There is still a lack of research on the interaction mechanisms between quercetin and OMSCs.

Although using antioxidants is a tried‐and‐true strategy for reducing excessive ROS, there are still problems that need our attention, such as the short half‐life, poor pharmacokinetics, and limited absorption of oral medicine.^[^ ^11^ ^]^ To raise the local concentration of medications and lengthen the duration of drug residence in periodontal tissue therapy and care, a strong local delivery system is needed.^[^ ^12^ ^]^ Bioglass is an ideal sustained‐release carrier for bioactive substances such as drugs and growth hormones. because of its high porosity and specific surface area. It can also offer a favorable surface for cell growth.^[^ ^13^ ^]^ A preliminary study demonstrated that mesoporous materials with high specific surface areas and pore sizes between 2–50 nm can effectively load drug molecules for long‐term controlled release.^[^ ^14^ ^]^

On the other hand, periodontal bone defects are often irregular, necessitating a material that can conform to the shape of the defect. For the repair of such deformities, injectable hydrogel material is suitable.^[^ ^15^ ^]^ It can provide uniform distribution in specific areas and is simple to inject into the subgingival pocket with its unusual shape. Its benefits also include simplicity in manufacturing, affordability, and minimal toxicity.^[^ ^16^ ^]^ Additionally, most hydrogels can degrade well without affecting new bone formation. Compared to conventional bone scaffolding materials, injectable hydrogels have the advantages of being less invasive and more movable, which can satisfy the demands of contemporary medicine for the accurate repair of irregular bone defects in periodontitis and minimally invasive surgery.^[^ ^17^ ^]^ However, when the drug is only enveloped in the hydrogel, it is confined only by physical interactions, which usually cause drug release to occur quickly and in large quantities rather than gradually.^[^ ^18^ ^]^ Therefore, it is worth exploring how to develop a sustained‐release system that can both achieve gradual drug release and solve the problem of irregular bone defect repair.

**Scheme 1:**
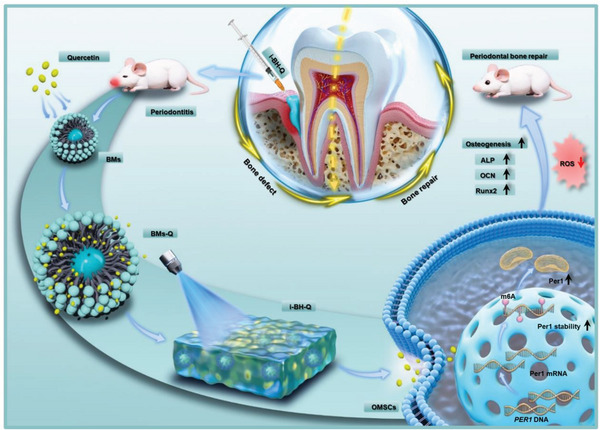
A diagram illustrating the mechanism by which the injectable quercetin‐loaded bioglass microsphere hydrogel system increases the expression of Per1 through quercetin‐mediated m6A modification of OMSCs, ultimately promoting the relief of the oxidative stress microenvironment and repair of periodontitis bone defects. BMs‐Q, quercetin‐loaded bioglass microspheres; BMs, bioglass microspheres; i‐BH‐Q, injectable quercetin‐loaded bioglass hydrogel.

Overall, we combined injectable hydrogel and bioglass as carriers to create a composite hydrogel that is simple to inject, light‐curable, and capable of drug loading and sustained release, and we demonstrated that the system may be used as an effective carrier for periodontal disease bone defect repair (**Scheme** **1**). This system was shown to increase the osteogenic differentiation of OMSCs by regulating Per1 expression. We also conducted an intensive study on the mechanism of interaction between this system and OMSCs to clarify whether this system regulates the expression of Per1 in OMSCs through m6A modification, attenuates oxidative stress damage in OMSCs, and promotes periodontal bone repair. The results showed that the hydrogel drug‐loading system in the present study is a promising therapeutic system for the treatment of periodontal disease bone defects.

## 2 Results and Discussion

### 2.1 Quercetin Alleviates the Effects of Oxidative Stress on OMSCs Differentiation, Proliferation, and Redox Levels

The involvement of mesenchymal stem cells is critical in the regeneration of periodontal tissue. OMSCs are the key cells in the healing process in terms of alveolar bone regeneration and blood supply irrigation in the diseased region following periodontal surgery. Inflammation, ROS, and other factors contribute to bone loss in periodontal disease. The differentiation and control of local stem cells is necessary for alveolar bone healing. To investigate the process of bone regeneration, it is vital to first investigate how quercetin controls local inflammation and regulates the mechanism of oxidative stress microenvironment. The effects of oxidative stress and oxidative stress therapy on cell stem differentiation were first observed in this section. In addition, we ensured that the cell survival rate was within 50–70% and chose 100 µm H_2_O_2_ for 30 min as the modeling standard for oxidative stress damage in OMSCs for subsequent experiments (Figure S1, Supporting Information).

**Figure 1:**
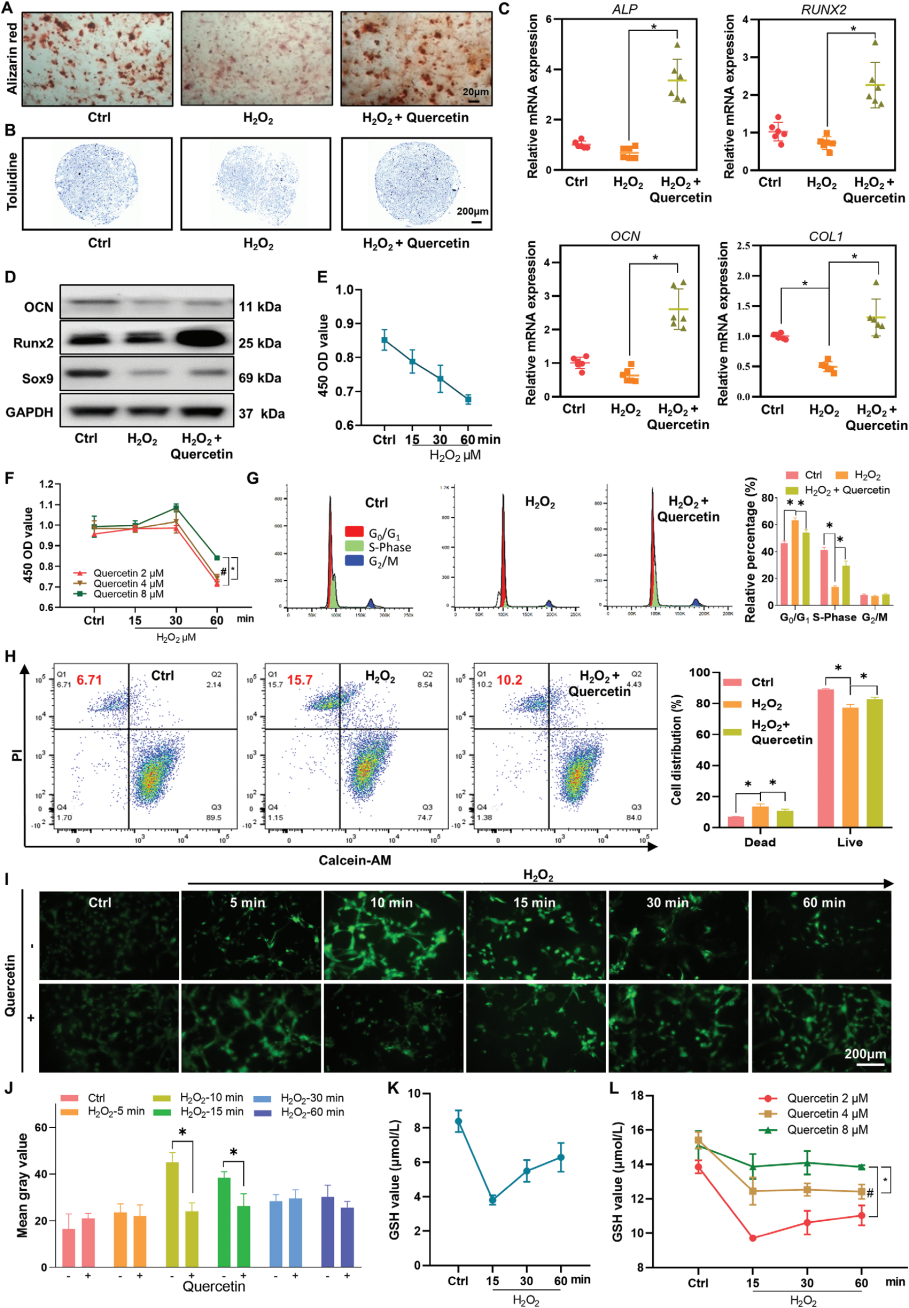
Quercetin mitigates the effects of the oxidative stress microenvironment on the differentiation, apoptosis, and oxidative stress levels of OMSCs. A, B) Alizarin red (A) and toluidine staining (B) of P2 generation OMSCs in the control group, oxidative stress group (100 µm H_2_O_2_ for 30 min, differentiation induction for 21 days), and quercetin treatment group (100 µm H_2_O_2_ for 30 min followed by 4 µm quercetin, differentiation induction for 21 days). C) RT‐PCR was performed to determine the expression of the *ALP, RUNX2, OCN*, and *COL1* genes in OMSCs in the control group, oxidative stress group (100 µm H_2_O_2_ for 30 min), and quercetin treatment group (100 µm of H_2_O_2_ for 30 min followed by 4 µm quercetin). D) Western blotting was used to identify the expression of OCN, Runx2, and Sox9 in OMSCs in the control group, oxidative stress group (100 µm H_2_O_2_ for 30 min), and quercetin treatment group (100 µm H_2_O_2_ for 30 min followed by 4 µm quercetin). E, F) The CCK‐8 assay was used to measure cell proliferation changes in OMSCs after 0, 15, 30, and 60 min of 100 µm H_2_O_2_ (E), as well as after 100 µm H_2_O_2_ followed by different concentrations (2 µM, 4 µM, and 8 µM) of quercetin (F). G, H) Flow assay for cell cycle (G) and live/dead cells (H) in control (PBS), oxidative stress (100 µm H_2_O_2_ for 30 min), and quercetin‐treated groups (100 µm H_2_O_2_ for 30 min followed by 4 µm quercetin for 2 h). *, *p* < 0.05. I, J) ROS levels (I) of OMSCs were measured and statistically analyzed (J) after 0, 5, 10, 15, 30, and 60 min of 100 µm H_2_O_2_, as well as after 100 µm H_2_O_2_ followed by 4 µm quercetin for 2 h. K, L) GSH levels (K) of OMSCs were measured and statistically analyzed (L) after 0, 15, 30, and 60 min of 100 µm H_2_O_2_, as well as after 100 µm H_2_O_2_ followed by different concentrations (2, 4, and 8 µm) of quercetin for 2 h. *n* ≥ 3, #, *, *p* < 0.05.

The results demonstrated that oxidative stress impaired the osteogenic and chondrogenic differentiation capabilities of OMSCs. Alizarin red staining was much lower than in the control group, but differentiation capacity was still strengthened somewhat by the addition of quercetin (**Figure** **1**A). Chondrocyte toluidine blue staining was weaker following induction and differentiation than in the normal control group. Similarly, quercetin deepened the staining of chondrocytes derived from OMSCs, showing that their chondrogenic differentiation capacity was recovered to some extent (Figure 1B). The results of the Oil red O staining also showed the ability of quercetin to promote its lipogenic differentiation (Figure S2, Supporting Information). The above results suggest that oxidative stress disrupts the differentiation stemness of OMSCs, while quercetin is able to repair the stemness damage caused by oxidative stress. At the gene level, the oxidative stress microenvironment decreased the expression of osteoblast‐related genes (*ALP, RUNX2, OCN*, and *COL1*), but the addition of quercetin enhanced the expression of osteoblast genes (Figure 1C). At the protein level, oxidative stress lowered the expression of bone‐related proteins (OCN, Runx2, and Sox9), while quercetin boosted the expression of homologous proteins (Figure 1D; Figure S3, *, *p* < 0.05, Supporting Information).

After that, the changes in cell activity were measured using a CCK‐8 assay, and the results showed that exposure to H_2_O_2_ alone resulted in a gradual decrease in cell activity with increasing action time (Figure 1E). The addition of quercetin rescued cell activity at the early stage of oxidative stress, but not at the late stage of oxidative stress (60 min). At the early stages of oxidative stress (15, 30 min), there was no significant difference in the effect of the three groups of quercetin (Figure 1F). We used flow cytometry to detect the OMSCs cell cycle under the same treatment circumstances. The proliferative phase (S‐Phase, green peak) cell count was considerably reduced in the H_2_O_2_‐treated group compared to the quercetin‐ and control‐treated groups, according to the results. On the other hand, oxidative stress stimulation leads cells to stagnate in the pre‐proliferative phase and decreases the number of cells entering the proliferative phase, as seen by the much higher number of cells in the G_0_ phase compared to the other two groups. Similarly, quercetin restores the oxidative stress‐induced alterations in the cell cycle and boosts the quantity of proliferating cells (Figure 1G). We also measured the percentage of dead and surviving cells after different treatments in order to obtain a more intuitive understanding of the effect of oxidative stress injury on the condition of OMSCs. The percentage of dead cells was considerably higher in the group treated with H_2_O_2_ than in the control group, according to the results. The percentage of dead cells dropped when quercetin was added (Figure 1H). The aforementioned findings indicate that oxidative stress raises the rate of apoptosis in OMSCs, yet quercetin can partially reverse this process. We further investigated the association between oxidative stress and quercetin based on the preceding findings. Dichlorofluorescin is oxidized to dichlorofluorescein in the presence of reactive oxygen species, resulting in a bright green fluorescent material that cannot pass through the cell membrane. Our measurement of intracellular ROS expression revealed that as H_2_O_2_ exposure duration increased, intracellular ROS production increased progressively, with the maximum expression in the early phase of action, i.e., at 10 and 15 min. As time passed, cells began to undergo apoptosis, resulting in a drop in ROS and a fall in cell number. Simultaneously, the addition of quercetin decreased excessive ROS generation in the early stages of cells while maintaining cell activity (Figure 1I,J, *, *p* < 0.05). A glutathione (GSH) assay was used to assess GSH expression in OMSCs after various treatments. GSH content can be measured colorimetrically at 405 nm. The results revealed that GSH expression was greatly reduced in the early stages of oxidative stress (Figure 1K), gradually increasing with the cell's response to the oxidative stress environment, but remaining significantly lower than that in the normal control group. The addition of quercetin can make GSH more responsive. GSH expression rises increased with increasing quercetin concentration, with substantials showing a significant difference (Figure 1L).

The findings in this section reveal that oxidative stress inhibits OMSCs differentiation, induces apoptosis, lowers cell proliferation, imbalances the redox level of the cells, and diminishes osteogenesis. In contrast, adding quercetin to cells subjected to oxidative stress damage will partially restore cell differentiation, maintain cell activity and redox levels, and promote osteogenic gene expression.

### 2.2 Quercetin Modulates the m6A Levels of Key Genes Involved in Cell Differentiation and Redox Processes in OMSCs to Control the Cell State

Periodontal disease and epigenetics interact with one another. As a key epigenetic change, m6A methylation and associated regulators have considerably contributed to our understanding of the biological activities of many cell types, such as stem cell maintenance, circadian rhythm modulation, and tumorigenic ability. m6A expression differs significantly in quercetin‐treated periodontitis, implying that epigenetic regulation of m6A plays a regulatory function in periodontal defect repair.^[^ ^7^ ^]^ However, the regulatory mechanism of m6A in periodontitis OMSCs is yet unknown.

**Figure 2:**
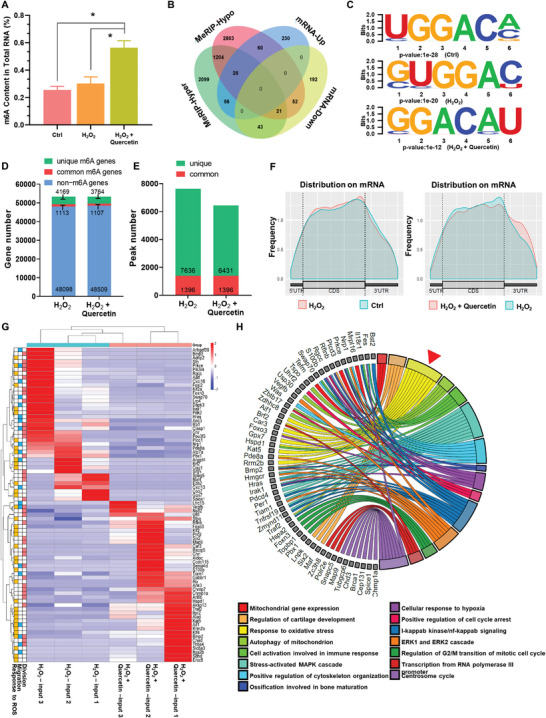
Quercetin alters the m6A characteristics of genes involved in cell differentiation and redox reactions in OMSCs. A) The m6A levels of total RNA of OMSCs in the control group, oxidative stress group (100 µm H_2_O_2_ for 30 min), and quercetin treatment group (100 µm H_2_O_2_ for 30 min followed by 4 µm quercetin) were determined. B) MeRIP‐seq was used to determine the m6A modification levels of RNAs in the three groups of OMSCs (control group, oxidative stress group (100 µm H_2_O_2_ for 30 min) and quercetin treatment group (100 µm H_2_O_2_ for 30 min followed by 4 µm quercetin)), and a Venn diagram from the MeRIP‐seq dataset of the latter two groups was constructed to display the overlap of the differentially m6A‐methylated genes and the differentially expressed genes. C) Motif analysis of peaks in the three groups of OMSCs was performed using Homer software. D) The number of m6A‐modified genes identified from the MeRIP‐seq dataset in the oxidative stress group and quercetin treatment group. E) Number of m6A peaks identified by m6A‐seq in the oxidative stress group and quercetin treatment group. F) Density curve showing the distribution of m6A peaks across the transcripts in the three groups. The transcript is divided into three parts, namely, 5′UTR, CDS, and 3′UTR. G) Heatmaps showing the expression profiles of the hyper‐up and hypo‐down differentially expressed genes in the three groups. The gradient of color from blue to red corresponds to changes in expression values from low to high. The functional classification corresponding to the differentially expressed genes is shown on the left side of the heatmap (red: cell division, blue: cell migration, yellow: response to ROS). H) The GO terms are visualized in a chord plot. CDS, coding DNA sequence; UTR, untranslated region. *n* = 3, *, *p* < 0.05.

As a result, in this section, we created an in vitro cellular oxidative stress model and added quercetin to it, collected RNA samples from the cells for an m6A expression assay, and discovered that the m6A levels of cells in the oxidative stress group and the group with the quercetin effect were elevated compared to the control group (**Figure** **2**A). Next, we used MeRIP‐seq to investigate the m6A mechanism of quercetin in modulating the oxidative stress status of OMSCs. The genes with changes in m6A modification were connected with the genes with changes in RNA expression levels to identify some critical genes associated with oxidative stress and quercetin treatment conditions for further research. Generally, there are four types of association analysis results: hypermethylated‐down (RNA hypermethylated‐RNA downregulation); hypermethylated‐up (RNA hypermethylated‐RNA upregulation); hypomethylated‐up (RNA hypomethylated‐RNA upregulation); and hypomethylated‐down (RNA hypomethylated‐RNA downregulation). Our Venn diagram analysis (Figure 2B) revealed that 56 of the differentially expressed genes had higher methylation levels as well as higher mRNA expression in the oxidative stress group than in the oxidative stress plus quercetin treatment group. On the other hand, 52 genes exhibited a simultaneous reduction in methylation and mRNA levels. There were 1204 genes with alterations in methylation and demethylation. In contrast, sixty genes had increased demethylation but reduced mRNA levels, and 43 genes had increased methylation but decreased mRNA levels. This finding implies that m6A alteration can influence gene mRNA expression levels via methylation and/or demethylation (Figure S4, Supporting Information).

Several investigations have found that m6A is mostly enriched for the motifs “RRACH” and “GGAC.” Accordingly, to confirm the reliability of the m6A‐seq assay and the specificity of the antibodies in the three groups of cells, we used Homer software to analyze the signature motifs in the three groups and discovered that specific m6A modifications were recognized in all three groups of cell samples (Figure 2C). We studied the m6A distribution patterns in both the oxidative stress group and the oxidative stress accompanied by the quercetin stimulation group using MeRIP‐seq. The results indicated that in the oxidative stress group, there were 4169 unique m6A‐modified genes and 7636 unique m6A peaks, while in the oxidative stress with quercetin group, there were 3764 unique m6A‐modified genes and 6431 unique m6A peaks (Figure 2D,E). A peak is a location with a high concentration of m6A alterations. Following the comparison of clean reads to the genome, the objective is to determine how these short sequences are enriched in the genome, a technique known as enriched area identification or peak calling. Exon‐based peak calling and differential RNA modification analysis were carried out using the program exomePeak (Figure 2E).

Peak annotation analysis is necessary after acquiring peaks to determine where these peaks are positioned in the gene structure and the general distribution characteristics. Based on the detected m6A peaks, a metagene plot was created to depict the distribution of each set of peaks on the RNA structure (Figure 2F). Heatmaps depicting the levels of expression of differentially expressed genes in three sets of RNA‐seq data were prepared. The functional classification corresponding to the differentially expressed genes is shown on the left side of the heatmap; in brief, red represents genes involved in cell division, blue represents genes involved in cell migration, and yellow represents genes involved in response to ROS (Figure 2G). Based on the results, the genes were subjected to GO analysis for computational enrichment, and GO chord maps were created to depict the reported gene enrichment pathways. The results of GO enrichment analysis showed that between the quercetin‐interacting group and the oxidative stress‐treated group, the differentially expressed genes were mainly enriched in genes related to the oxidative stress pathway (red triangles) and genes related to the osteogenic differentiation pathway (Figure 2H). The results of the enrichment analysis of the three groups simultaneously by Kmeans showed that the differential genes among the three groups were also enriched in oxidative stress‐related pathways such as HIF‐1α (Figure S5, Supporting Information).

### 2.3 Quercetin Increases the Level of m6A Modification and Expression of the Per1 Gene in OMSCs and Influences Cell Osteogenic Development

**Figure 3:**
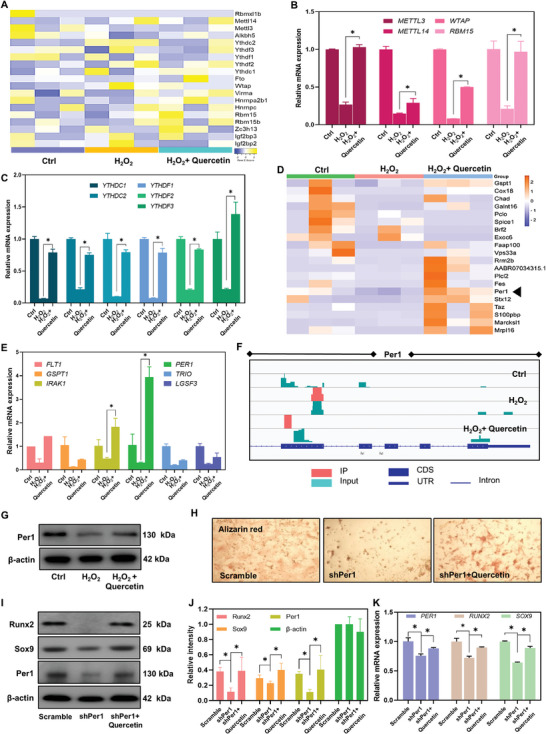
Quercetin enhanced the m6A modification level and the expression level of the Per1 gene in OMSCs, whereas inhibiting the Per1 gene lowered the expression of osteogenesis‐related genes in OMSCs. A) Heatmap analysis of the expression changes of key m6A modification enzymes in OMSCs in the three groups with different treatments. B, C) In vitro RT‐PCR validation of key m6A methylation and reading protein enzymes in OMSCs from the three groups. D, E) Heatmap analysis (D) and in vitro RT‐PCR validation (E) of differentially altered osteogenic or oxidative stress‐related genes were performed according to the MeRIP‐seq dataset from the three groups. F) The m6A peak visualization according to the MeRIP‐seq dataset of Per1 transcripts in OMSCs was made by IGV software. G) WB was used to determine the expression of Per1 in OMSCs from the three groups. H) Alizarin red staining of OMSCs subjected to osteogenesis‐induced differentiation for 21 days after inhibition of Per1 or after inhibition followed by quercetin. I, J) WB determination and statistical analysis of osteogenesis‐related protein expression in OMSCs after in vitro knockdown of Per1 as well as after inhibition followed by quercetin. K) RT‐PCR detection of *PER1*, *RUNX2*, and *SOX9* after in vitro knockdown of Per1 and 48 h post‐inhibition treatment with quercetin. *n* ≥ 3, *, *p* < 0.05.

Based on MeRIP‐seq data, we performed heat map analysis on the expression of enzymes that are important in m6A modification, such as methylases, demethylases, and reading proteins, to further study how oxidative stress and quercetin influence the cellular state via the m6A modification pattern (**Figure** **3**A). The results demonstrate that the expression levels of certain mutated enzymes were altered in the three groups in opposing directions. *METTL3* expression of m6A in OMSCs, for example, was inhibited by the influence of a peroxygenated environment, but *METTL3* expression was increased and substantially different with a significant difference after quercetin treatment (Figure 3B). In the meanwhile, we discovered that oxidative stress injury in OMSCs decreased the expression of important reading proteins that are now known, such as members of the YTHDC and YTHDF families. This was followed by a notable increase brought on by the addition of quercetin (Figure 3C). Based on the MeRIP‐seq assay, we discovered that the m6A modification levels of numerous genes differed before and after quercetin administration (Figure 3D). By combining these results with in vitro validation, the mRNA level of *PER1* was shown to be considerably increased, which was consistent with the sequencing results (Figure 3E). The Per1 peak location data were put into the IGV display, and Figure 3F depicts the portion of the site where the m6A mutation occurred. Per1 has been reported to be involved in regulating metabolic homeostasis in oxidative stress microenvironments. Accordingly, we employed western blotting (WB) to confirm our findings. Per1 expression was shown to be considerably higher following quercetin administration (Figure 3G; Figure S6, *, *p* < 0.05, Supporting Information). After explicitly knocking down Per1 or after knocking down and adding quercetin, we began osteogenic differentiation in OMSCs. The findings demonstrated that OMSCs' capacity to develop osteoblastically was impacted by Per1 knockdown, as indicated by a decrease in the amount of calcium nodules. On the other hand, quercetin supplementation following Per1 knockdown boosted OMSCs' capacity to differentiate into osteoblasts (Figure 3H). As can be seen in the WB (Figure 3I,J) and RT‐PCR (Figure 3K) validation, after the knockdown of the Per1 gene in vitro, the expression of osteogenesis‐related genes decreases. However, the addition of quercetin co‐culture after knockdown again increased the expression of osteogenesis‐related genes in OMSCs. This result suggests that the reduction of the Per1 gene attenuates the expression of osteogenic genes in OMSCs, while quercetin can still restore the osteogenic ability of OMSCs to a certain extent on this basis (*, *p* < 0.05).

This section primarily focuses on the analysis and validation of methylation‐related enzymes by bioinformatics analysis results combined with in vitro cytological validation. First, the enzymes related to m6A modification that cause cells to undergo m6A modification were analyzed and validated. The methylation‐related enzymes were found to show different changes in the oxidative stress group and the quercetin‐treated group. According to data screening and validation, the Per1 gene differed among groups in terms of RNA modification levels as well as gene and protein expression levels, and silencing the Per1 gene in vitro affected the ability of OMSCs to develop into osteoblasts. This finding implies that Per1 may function as a quercetin target to control the osteogenic differentiation status of OMSCs, which serves as a reference for the in‐depth examination of quercetin's mechanism of action.

### 2.4 Preparation and Characterization of Bioglass and Injectable Hydrogel

According to the aforementioned experimental findings, we preliminarily confirmed quercetin's ameliorative effect on the oxidative stress state of OMSCs and preliminarily explored its molecular mechanism in vitro to support the ability of cells to differentiate into osteoblasts by mediating the m6A modification of Per1. Further research is necessary to determine whether quercetin can eventually contribute to bone healing in a periodontal disease model that includes oxidative stress status and in vivo bone loss. However, it is also necessary to consider that there are challenges involving the local action of quercetin in vivo, including the rapid local release, the tendency toward uneven local concentration distribution, and the challenge of local fixation. To address the aforementioned issues, an appropriate medication delivery system must be built.

**Figure 4:**
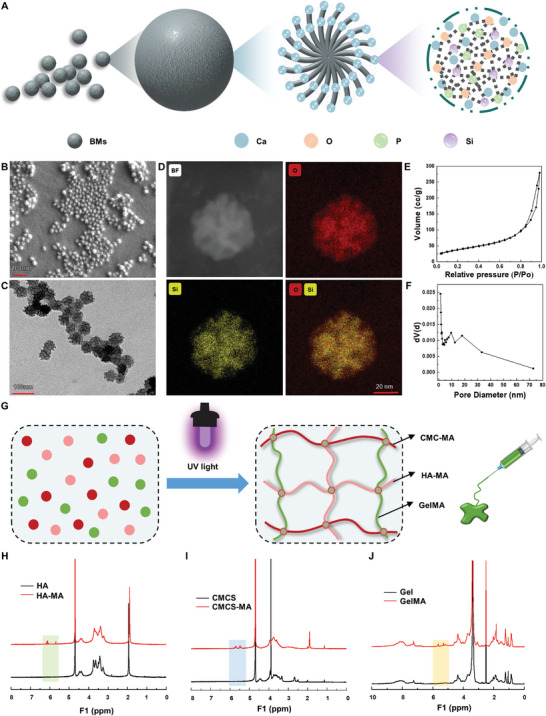
Synthesis of bioglass and injectable hydrogel. A) Schematic diagram of the preparation of bioglass (BMs). B–D) SEM, TEM, and EDS micrographs of BMs. E, F) N_2_ adsorption–desorption isotherms (E) and the corresponding pore size distribution curve (F) of the BMs. G) Schematic diagram of the preparation of injectable hydrogels. H–J) ^1^HMR spectra of HA‐MA, CMCS‐MA, and GelMA.

First, based on inspiration from our earlier study, we constructed a bioglass to achieve localized gradual release. The osteogenic effect of bioglass and its ability to degrade in vivo with good biocompatibility have been preliminarily demonstrated. In this work, bioglasses were created utilizing organic self‐assembly and sol–gel procedures in a water–oil biphasic layered reaction system. The created bioglasses had good radiomorphology and wide pores with a diameter of ≈50 nm, allowing the loading and delayed release of quercetin (**Figure** **4**A). Small amounts of calcium and phosphorus pentoxide in bioglass can to some extent facilitate the deposition of hydroxyapatite, which increases the material's osteogenic properties and makes it more appropriate for use in bone repair. From scanning electron microscopy (SEM), transmission electron microscopy (TEM) in Figure 4B,C, as well as energy dispersive spectrometer (EDS) analysis in Figure 4D of the bioglass microspheres (BMs), it can be seen that BMs have a regular spherical morphology, uniform particle size distribution, radial structure, and compactness, specifically a radial mesoporous interior structure. In Figure S7A (Supporting Information), we can clearly see that the bioglass after loading the quercetin drug shows pale yellow in color, while the pure bioglass is white, indicating that quercetin was successfully loaded into the pores of the bioglass. In addition, in Figure S7B (Supporting Information), the morphology of the quercetin‐loaded bioglass still showed a regular spherical shape and uniform pores, indicating that the morphology of the bioglass would not be changed by the loading of the small‐molecule drug (quercetin) and that the drug‐loading system had good stability. The N_2_ adsorption–desorption technique was employed to characterize the surface and pores of the bioglass. As shown in Figure 4E,F, the adsorption–desorption curve is compounded with a type IV curve, indicating that the bioglass exhibits a mesoporous structure. At high relative pressures, the curves show a clear adsorption hysteresis loop, indicating that the pore structure of the bioglass is irregular. The calculated specific surface area of the bioglass is 140.93 m^2^ g^−1^, and the distribution of pore size is in the range of 1.42–10.1 nm, which is favorable for drug loading.

After attaining gradual drug release, it was necessary to address local drug distribution and immobilization within the body. Therefore, we chose an injectable hydrogel with good fluidity as the carrier and distributed the drug‐carrying bioglass uniformly in the hydrogel. As shown in Figure 4G, the reaction of gelatin, hyaluronic acid, and carboxymethyl chitosan with methacrylic anhydride resulted in a final polymer with a double‐bond structure. The injectable hydrogel forms under the action of UV light and consists mainly of methacrylated gelatin (GelMA), methacrylated hyaluronic acid (HA‐MA) and methacrylated carboxymethyl chitosan (CMCS‐MA). The successful fabrication of GelMA, HA‐MA, and CMCS‐MA was confirmed by ^1^H‐NMR (Figure 4H–J).

### 2.5 Injectability, Morphology, and Drug‐Blocking Analysis of Hydrogels

Since this material involves curing under UV light, we also verified the degradation of quercetin under UV light in the previous period in order to determine the sensitivity of quercetin used in this study to UV light and to avoid the effect of UV light on the pharmaceutical properties of quercetin. Quercetin is a small‐molecule drug, it is usually more stable in its molecular structure. The structural formula of the drug is shown in Figure S8A (Supporting Information). In addition, we also carried out UV–vis absorption spectroscopy, and the results showed that the working concentration of the quercetin solution we used did not undergo significant degradation even under prolonged UV illumination (30 min). Therefore, we believe that the concentration and function of quercetin will not be affected within 1 min of our UV illumination (Figure S8B, Supporting Information).

**Figure 5:**
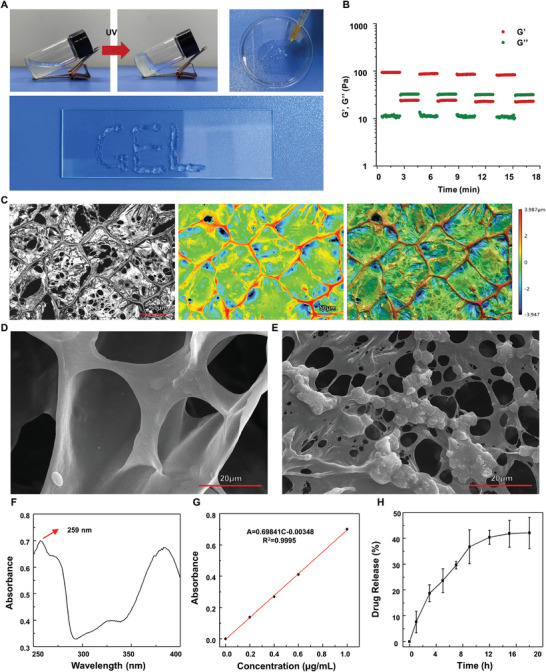
Preparation and characterization of an injectable bioglass hydrogel (i‐BH) drug delivery system. A) Injectability of hydrogels (letter “GEL”). B) Continuous step‐strain measurements were applied to the hydrogel at an angular frequency of 1 Hz, and oscillatory strains were switched from *γ* = 1–200%. C) Confocal topography of the hydrogel. D) SEM images of hydrogel and E) bioglass‐loaded hydrogel. F) UV‐vis absorption curve of quercetin. G) The standard curve of the drug with different concentrations and release profiles H) of drugs.

As shown in **Figure** **5**A, the precursor solution turns into a hydrogel under UV irradiation, and the hydrogel can be smoothly injected through a 26‐gauge needle to write “GEL,” indicating that the hydrogel has good injectable properties (Movie S1, Figure S9. Supporting Information). The immediate self‐repair capacity of the injectable hydrogel was then investigated by cyclic oscillatory strain sweeps between 1% and 200% at a continuous frequency of 1 Hz. As shown in Figure 5B, 200% strain led to transient disruption of the hydrogel network. However, the microstructure was completely reconstructed within 2 min as G' rapidly returned to the normal value. The above experimental results indicate that the hydrogel exhibits effective structural recovery during step changes in strain.

Scanning electron microscopy and laser confocal microscopy analysis revealed that the hydrogels have a homogeneous interconnected porous structure (Figure 5C,D). Moreover, the successful loading of BMs into the matrix of the hydrogel is shown in Figure 5E. In addition, it is obvious that BM is uniformly distributed in the composite hydrogel by the Si element in the EDS image (Figure S10, Supporting Information). As shown in Figure 5F, the drug had a strong UV absorption peak at 259 nm. In addition, a fitted linear equation (Figure 5G) was used to quantitatively analyze the sustained‐release effect of the drug‐loaded injectable hydrogel. The drug release results (Figure 5H) indicated that ≈42.07% of the drug (quercetin) was released from the hydrogel after 18 h. Ultimately, we combined the aforementioned BMs, hydrogels, and quercetin to construct an injectable quercetin‐loaded bioglass hydrogel system (i‐BH‐Q) for subsequent biological studies.

### 2.6 The Injectable Quercetin‐Loaded Bioglass Hydrogel Promotes the Osteogenesis of OMSCs Dependent on Per1

**Figure 6:**
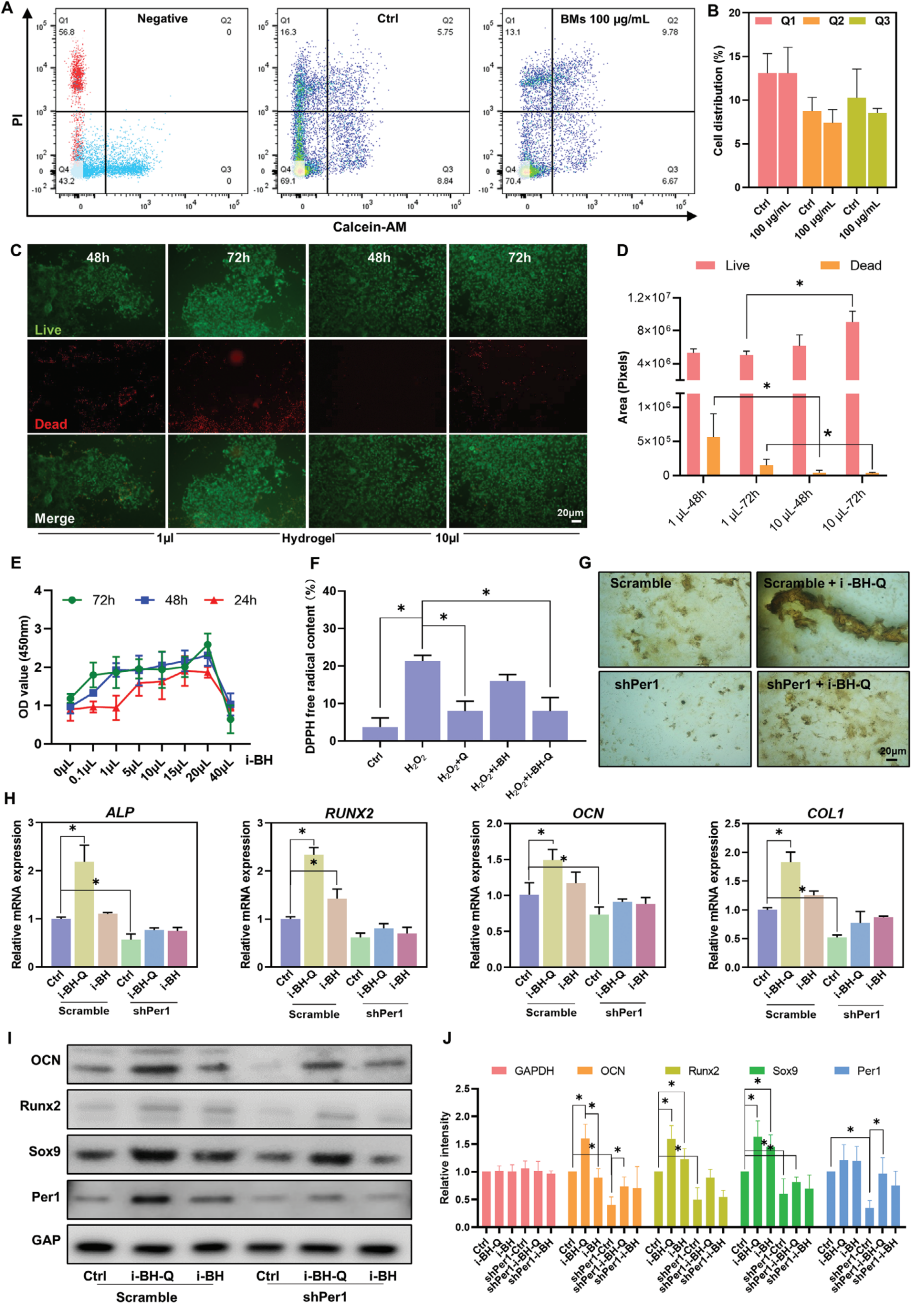
The i‐BH‐Q system has good biosafety and promotes the osteogenic differentiation of OMSCs dependent on Per1. A, B) OMSCs were added to autoclaved and sterilized bioglass (100 µg mL^−1^). Flow cytometry was used to evaluate the cell state after 48 h of coculture. C, D) Live/dead assay was performed to detect the cellular status of OMSCs after coculture with different concentrations (1 µL and 10 µL) of injectable hydrogels for different periods (48 and 72 h). E) i‐BH of varying concentrations was added to the cell culture medium (0, 0.1, 1, 5, 10, 15, 20, and 40 µL of 4% hydrogels were added to 100 µL α‐MEM). The cytotoxicity of the hydrogels was detected by CCK‐8 after cocultivation with the OMSCs for 24, 48, and 72 h. F) Detection of DPPH levels of cells in the control group, H_2_O_2_ group (100 µm action for 30 min), H_2_O_2_ + quercetin group (100 µm action for 30 min, 4 µm quercetin action for 2 h), H_2_O_2_ + i‐BH group (100 µm action for 30 min, 20 µL/100 µL of i‐BH action for 72 h), H_2_O_2_ + i‐BH‐Q group (100 µm action for 30 min, 20 µL/100 µL of i‐BH‐Q for 72 h). G) After transfected with scramble vector or shPer1 plasmid, 20 µL/100 µL of i‐BH‐Q or i‐BH was given to co‐cultivate for 72 h, along with the results of alizarin red staining after 21 days of osteogenic induction. H–J) RT‐PCR (H) and WB (I, J) detection of osteogenesis‐related gene expression in primary OMSCs and Per1‐specific inhibited OMSCs in the presence of i‐BH‐Q or i‐BH system for 48 h. *n* ≥ 3, *, *p* < 0.05.

The cytocompatibility of the i‐BH‐Q system with OMSCs was examined from different angles in this section. First, we tested the biosafety of BMs using flow cytometry. The results revealed that the proportion of early apoptotic cells was slightly lower in the 100 µg mL^−1^ experimental group than in the control group, while the percentage of late apoptotic cells was slightly greater, although neither was significantly different (**Figure** **6**A,B). This result suggested that at the proper concentration of bioglass material, OMSCs activity was good, and the bioglass did not cause evident cytotoxicity to OMSCs but instead had high biocompatibility.

Next, we observed the cellular status of OMSCs inoculated on the hydrogel by dead and live cell staining. OMSCs inoculated on hydrogel clearly had a high viability rate, and increasing the hydrogel concentration (from 1 µL/100 µL to 10 µL/100 µL) also resulted in improved OMSC proliferation and cell status. This was proven by the fact that after 72 h of coculture of OMSCs with the hydrogel extract, the number of live cells in 1 µL/100 µL hydrogel extract concentration was significantly less than that in 10 µL/100 µL concentration of the hydrogel extract (Figure 6C), and the number of dead cells in contrast (also shown in the results of the cocultivation for 48 h), was significantly higher in this group, and the differences were significant (Figure 6D, *, *p* < 0.05). The results showed that at low concentrations of hydrogel, the cells still have normal apoptosis. As the hydrogel concentration increased, its protective effect on the cells became more obvious, the cell activity increased, and therefore the cell regulation was reduced. All of the aforementioned findings show that the hydrogel and bioglass we employed in our investigation are both biocompatible and suitable for use in future in vivo experiments.

Besides, we investigated the biocompatibility of an injectable bioglass hydrogel (i‐BH). The results showed that the hydrogels were more effective than the blank control group. The experimental results revealed that compared to the blank control group, the OD value of OMSCs increased progressively as the hydrogel concentration increased, but at 40 µL/100 µL, the hydrogel displayed evident toxicity, inhibiting cell growth. Moreover, the cells cocultured for 48 and 72 h were in overall better condition than those cocultured for 24 h. These results showed that the longer the cells were cultured with this hydrogel at the optimal concentration of 20 µL/100 µL, the better the cell proliferation status was observed (Figure 6E).

Furthermore, we analyzed the alterations in free radical composition inside OMSCs to explore the materials' capacity to scavenge oxidized free radicals within cells in an in vitro setting. The findings demonstrated that the H_2_O_2_ group's cell content of 2, 2‐diphenyl‐1‐picrylhydrazyl (DPPH) was much higher than that of the control group, approaching five times that of the latter. As opposed to this, the DPPH content with the addition of quercetin was twice as high in the control group but significantly lower in the group that had experienced oxidative stress. A small but statistically insignificant reduction in the free radical content was observed in the i‐BH treated group, while the free radical content in the i‐BH‐Q treated group was substantially lower than that of the oxidative stress‐injured group and nearly identical to that of the quercetin group (Figure 6F).

Subsequently, OMSCs underwent osteogenic differentiation after transfection with shPer1 and its scramble vector. The outcomes demonstrated that the addition of i‐BH‐Q markedly increased the osteogenic capacity than in the empty vector control group. Following Per1 inhibition, the osteogenic potential of OMSCs was diminished. However, it was subsequently markedly increased upon the addition of i‐BH‐Q. Those findings revealed that OMSCs' osteogenic differentiation capacity was decreased with Per1 inhibition, while i‐BH‐Q was able to partially restore the above negative effect (Figure 6G).

Having confirmed the good biosafety of the slow‐release quercetin hydrogel system, we further explored the intrinsic relationship of this hydrogel system with Per1 and osteogenesis‐related genes in OMSCs. Both RT‐PCR (Figure 6H) and WB (Figure 6I,J) results showed that the quercetin‐loaded hydrogel system was able to increase the expression of Per1 and osteogenic genes in OMSCs compared to the control group. This is more consistent with our previous results. The hydrogel system without quercetin loading also slightly promoted the expression of the Per1 gene and cellular osteogenic genes. However, when the expression of the Per1 gene in OMSCs was inhibited, the osteogenic genes in OMSCs were significantly reduced, and the addition of the quercetin‐loaded hydrogel system slightly alleviated the inhibition of Per1, while the osteogenic genes in the hydrogel system without quercetin showed almost no improvement.

In recent years, natural compounds such as quercetin have been found to act as effective senescence agents. Based on preclinical and early clinical data from senescence agent studies, taking quercetin appears to be effective in preventing or mitigating cancer formation. However, most of the current studies have focused mainly on the role of quercetin in the treatment of tumor cellular senescence.^[^ ^19^ ^]^ Therefore, we validated the relationships between quercetin and cellular senescence and between i‐BH‐Q and senescence of OMSCs in this study using senescence‐associated β‐galactosidase (SA‐β‐gal) staining. The results showed that after oxidative stress treatment, the number of senescent cells in OMSCs was greatly increased compared to normal cells, as evidenced by the activation of SA‐β‐gal. There was a significant increase in the number of blue cells, and the cells underwent irreversible growth arrest and became larger in size. The addition of quercetin after H_2_O_2_ treatment significantly reduced cellular senescence, with a decrease in the number of SA‐β‐gal positive blue cells and a lighter coloration. The addition of i‐BH alone based on H_2_O_2_ injury reduced cellular senescence to some extent, but the effect was not as good as that of the quercetin‐treated group. Finally, we added our prepared i‐BH‐Q to oxidative stress‐injured OMSCs, which had the best anti‐aging effect in this group (Figure S11, Supporting Information).

This section's findings demonstrate the good cytocompatibility of injectable hydrogels and bioglass. In the meantime. The outcomes demonstrated the good scavenging potential of the i‐BH‐Q system against cellular free radicals. Simultaneously, osteogenic differentiation of OMSCs was significantly promoted by the i‐BH‐Q system. On the other hand, the i‐BH‐Q system could still induce osteogenic differentiation after Per1 gene expression was inhibited, though the promotion degree was comparatively lower than in normal cells.

### 2.7 The Injectable Quercetin‐Loaded Bioglass Hydrogel System Promotes the Repair of Bone Defects in Periodontitis in Rats

**Figure 7:**
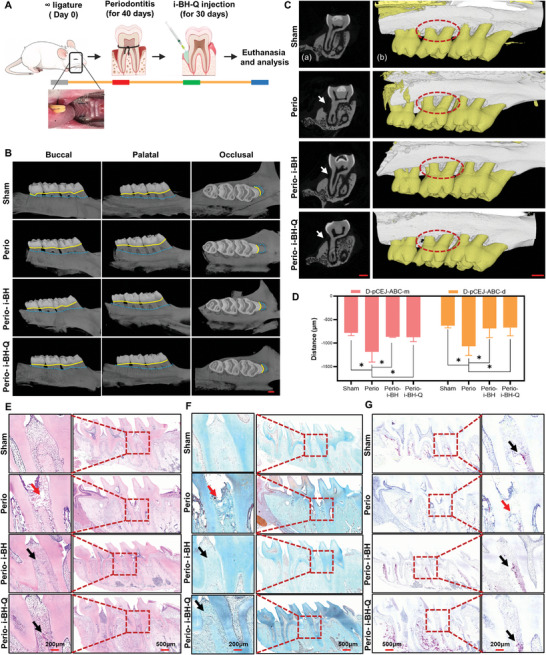
The injectable quercetin‐loaded bioglass hydrogel system can effectively promote the repair of bone defects in periodontitis in rats. A) Diagram of the in vivo validation of the effect of i‐BH‐Q on periodontitis bone defect repair. A periodontitis bone defect model was constructed by silk ligation in rats. i‐BH or i‐BH‐Q was added to the defect area after 40 days of model construction. Rats were sacrificed 30 days after surgery, and maxillary tissues were taken for analysis. B) The buccal, palatal, and occlusal surfaces of the maxillary molars of rats in the control, periodontitis, and periodontitis treated with i‐BH or i‐BH‐Q groups are shown separately, with the blue dashed line showing the position of the alveolar ridge and the yellow solid line marking the enamel‐dentin boundary. C) Palatal views of maxillary molars in the four groups. a) Distance between cementoenamel junctions to the alveolar bone crest (CEJ‐ABC) on the palatal side of the coronal surface of the second molar (white arrows). b) CEJ‐ABC distance (red dotted circle) in the palatal view of the maxillary 3D reconstruction. Scale bar: 1 mm. D) The distance from the CEJ to the ABC of the maxillary second molars was analyzed based on the micro‐CT results. *n* = 4, *, *p* < 0.05. E–G) Representative H&E (E), safranin solid green blue (F), and TRAP (G) staining images of each group. Sham, the control group; Perio, the periodontitis group; Perio‐i‐BH‐Q, the restored group.

Based on the above in vitro cytological test results, we concluded that the i‐BH‐Q system could be used for periodontitis bone defect repair in rats because of a variety of properties such as the antioxidant and anti‐inflammatory effects of quercetin, the fluidity and degradability of the hydrogel, and the slow‐release nature of the microsphere system. Therefore, a rat periodontitis bone defect model was created to test the osteogenic effect of the composite in periodontitis bone defect repair in vivo. This model simulated the clinical periodontitis formation process by forming localized plaque and food retention in the neck of the rat's maxillary second molar through silk ligation, forming an alveolar bone inflammation and oxidative stress microenvironment in the neck of the molar, and gradually causing resorption breakage. Injectable bioglass hydrogel or quercetin‐loaded bioglass hydrogel was injected into the bone defects after the model was constructed, and the alveolar bone repair was observed (**Figure** **7**A). Maxillary samples from each group were collected, fixed with paraformaldehyde, and examined using micro‐CT. The differences in the distances between the molar cementoenamel junctions (CEJ, Figure 7B, yellow solid line) and the alveolar bone crest (ABC, Figure 7B, blue dotted line) in the three groups could be seen from the buccal, palatal, and occlusal perspectives. The distance between the two lines from the CEJ to the ABC increased much more in the periodontitis group (Perio) than in the control group (Sham), demonstrating the successful construction of an alveolar bone defect model.

Either i‐BH or i‐BH‐Q was injected into the periodontitis bone defect, and the distance between the two lines shrank to closely resemble that of the control group (Figure 7B). The pictures were examined further in 2D coronal view (Figure 7C,a) and 3D reconstruction (Figure 7C,b). The mesial (D‐pCEJ‐ABC‐m) and distal (D‐pCEJ‐ABC‐d) CEJ‐ABC distances from the palatal side of the second molar were quantified in four groups in the coronal position. The CEJ‐ABC distance in the periodontitis group increased statistically significantly (Figure 7D, *, *p* < 0.05) due to bone abnormalities (Figure 7C, white arrow). The height of the alveolar ridge apex was significantly reestablished in the restored groups (Perio‐i‐BH and Perio‐i‐BH‐Q groups), despite the presence of some periodontal pockets (Figure 7C, white arrow). The restored groups showed significant differences from the periodontitis group (Figure 7D, *, *p* < 0.05). Differences in alveolar ridge apex height between the four groups were similarly visible on the palatal side of the 3D reconstruction images (Figure 7C, red dotted circle).

Rat maxillary molar tissue was removed and stained histologically. Calcium salt granules and nuclei are seen as dark blue in H&E staining The cytoplasm was stained by eosin in varying shades of pink to peach, collagen fibers in light pink, and elastin fibers in bright pink. Both hematoxylin‐eosin staining (H&E) and saffron solid green staining demonstrated significant retraction of the alveolar bone and degradation of the periodontal tissues in the periodontitis bone defect groups (Figure 7E,F, red arrows). The Perio‐i‐BH and Perio‐i‐BH‐Q groups, on the other hand, showed new bone trabeculae and an increase in the height of the alveolar ridge crest (Figure 7E,F, black arrows).

Anti‐tartaric acid phosphatase (TRAP) staining shows a burgundy coloring of the periosteal or cytoplasmic component of osteoclasts on a light blue or green background. There were significant burgundy highlights in the control, Perio‐i‐BH, and Perio‐i‐BH‐Q groups (Figure 7G, black arrow), indicating sufficient blood flow to the alveolar bone and active osteogenesis. In contrast, the periodontitis bone defect group had a lower percentage of burgundy color, indicating an imbalance in bone tissue regeneration (Figure 7G, red arrow).

**Figure 8:**
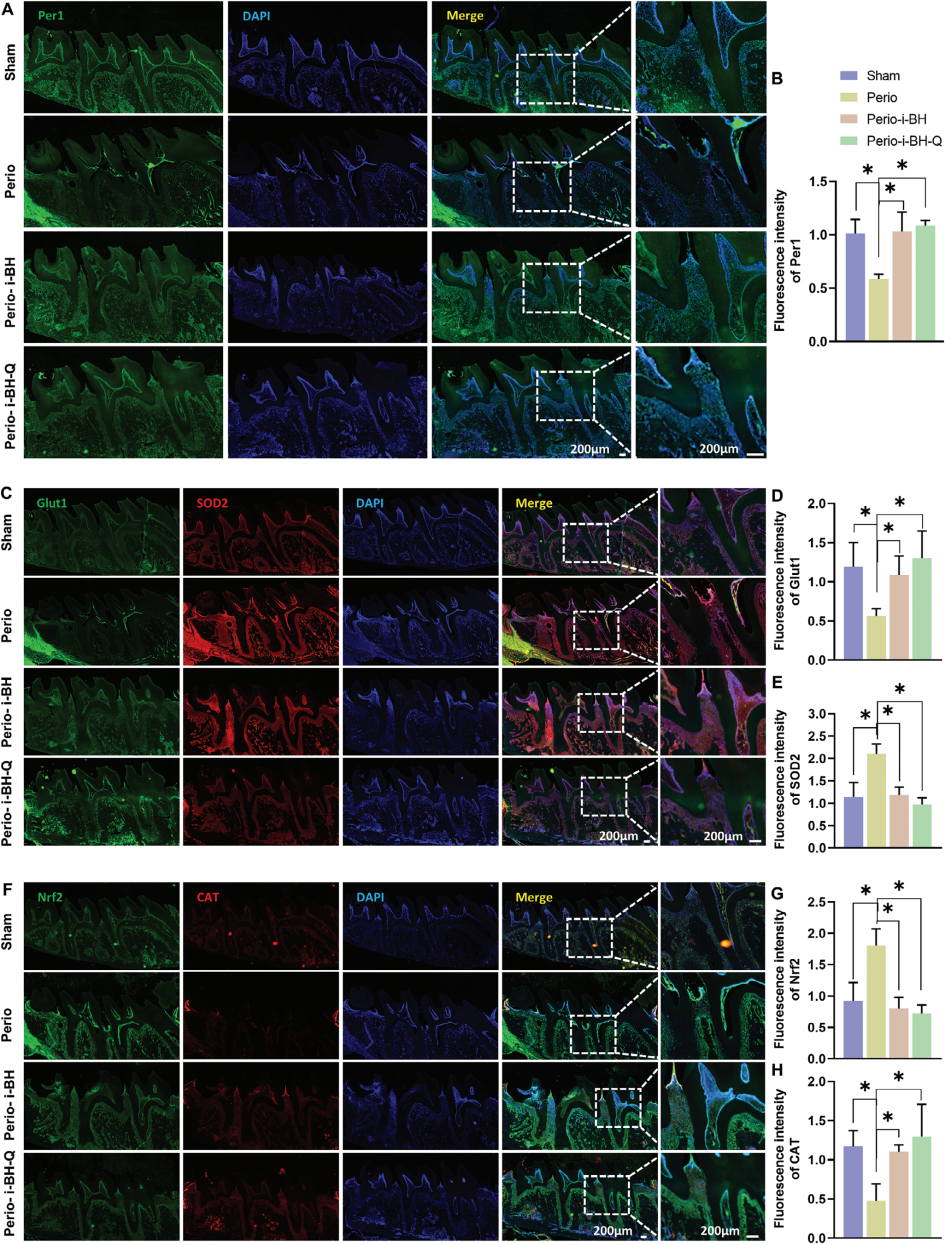
i‐BH‐Q enhances Per1 expression and alleviates the oxidative stress microenvironment in bone defect tissues of rat periodontitis. A–H) Immunofluorescence staining images and statistical analysis of Per1 A, B), Glut1 and SOD2 C–E) and Nrf2 and CAT F–H) in the maxillary molars of rats from control, periodontitis, and periodontitis groups treated with i‐BH or i‐BH‐Q. Sham, control group; Perio, periodontitis group; Perio‐i‐BH, bioglass hydrogel group; Perio‐i‐BH‐Q, quercetin‐loaded bio‐glass hydrogel group. *n* = 4, *, *p* < 0.05.

Furthermore, we investigated the degree of Per1 expression in rat alveolar bone tissue. It is evident that the periodontitis group had considerably lower Per1 expression than the control group. Nevertheless, Per1 expression rose in response to i‐BH or i‐BH‐Q treatment for periodontitis. Our in vitro cytological findings, which show that oxidative stress causes tissue cells to express less Per1, are in line with the outcomes of this animal experiment. Furthermore, adding i‐BH‐Q could increase Per1 expression while repairing the damage caused by oxidative stress at the same time (**Figure** **8**A,B).

Hyperoxic environments have been demonstrated to reduce the synthesis and transport of glucose transporter protein 1 (Glut1). ROS and inflammatory chemicals like necrosis factor‐α (TNF‐α) and lipopolysaccharide (LPS) boost superoxide dismutase 2 (SOD2) levels, which protect cells and regulate oxidative stress (Figure 8C–E). NF‐E2‐related factor 2 (Nrf2) is a transcription factor that controls the antioxidant stress response and protects cells from the harmful effects of oxidative stress. Catalase (CAT) is a key enzyme in the body's antioxidant defense mechanism (Figure 8F–H). To investigate the effect of the hydrogel system on the oxidative stress microenvironment in periodontitis, we labeled the aforementioned markers in alveolar bone tissue using immunofluorescence. Results show that the expression of Glut1 and CAT decreased while the expression of Nrf2 and SOD2 increased significantly in the periodontitis bone defect group. This implies that oxidative stress exists in the periodontal tissues of the periodontitis group, and it appears the organism produces antioxidants to counteract this response. Compared to the periodontitis group, the Perio‐i‐BH and Perio‐i‐BH‐Q groups had lower levels of the aforementioned index. That is, Glut1 and CAT expression increased while Nrf2 and SOD2 expression decreased, indicating that oxidative equilibrium had been restored.

In fact, the impact of quercetin mixed with biomaterials in promoting bone repair in various disorders has been partially investigated. Xin et al. initially showed that a hydrogel membrane composed of GelMA, bioglass, and recombinant human bone morphogenetic protein‐2 (BMP‐2) may exhibit good long‐term osteogenic properties and stimulate bone tissue regeneration.^[^ ^20^ ^]^ Further, scholars placed human umbilical cord MSCs‐derived sEVs onto 3D‐printed bioglass scaffolds coated with hydrogel. The substance promoted calcium deposition and endothelial network development.^[^ ^21^ ^]^ According to Wu et al., the bioglass hydrogel treated with lithium promoted cell division, directly induced osteogenesis.^[^ ^22^ ^]^ After that, Wang et al. produced bioglass with 8 nm pores in which they embedded BMP‐2 together with a GelMA hydrogel material that contained dasatinib and quercetin. They found that the substance stimulated MSC osteogenic growth and improved osteoporotic bone repair.^[^ ^23^ ^]^ Han et al. created ultrasmall, naturally liganded iron–quercetin nanoparticles with exceptional anti‐inflammatory and antioxidant effects.^[^ ^24^ ^]^ However, their iron–quercetin nanoparticles are in liquid form, making them more suitable for closed habitats like as joint cavities and possibly less suitable for open defect areas such as periodontal bone defects.

In this study, we used a quercetin‐based topical delivery strategy to treat periodontitis, a bone defect disease. We aimed to address the feasibility and biosafety of quercetin for oral periodontal medical purposes. We efficiently loaded quercetin, a small‐molecule drug, into an injectable bioglass hydrogel by a one‐step method, integrating the excellent functionality of all three elements to achieve versatility. Unlike systemic drugs that function through the bloodstream, the suggested hydrogel drug delivery technology can be used directly for localized oral drug administration to treat periodontal bone defect diseases. As a result, it is possible to avoid the previously mentioned disadvantages of the hydrogel's monofunctionality and immobilization of other bioglasses, as well as the ineffective systemic injection of quercetin.

From one perspective, we highlight several important advantages of injectable bioglass hydrogel delivery systems as topical periodontal medications, including i) protection of the loaded medication from residing in the moist and fluid environment of the oral cavity, ii) long‐lasting antioxidant and bone‐enhancing properties; and iii) excellent biosafety. From another perspective, we also explored the mechanisms of action of the above systems, including i) quercetin is an effective antioxidant; ii) quercetin modulates the m6A modification of Per1 to repair oxidative stress damage; and iii) quercetin‐loaded bioglass hydrogel is antioxidant, bone‐contributing, and repairs the periodontal oxidative stress microenvironment. As a historical drug resource, quercetin has intriguing properties that could open new avenues for more innovative diagnostic and therapeutic options. However, continued exploration is needed to realize the future clinical translation of quercetin drug‐carrying systems.

## 3 Conclusion

In conclusion, this study developed a quercetin‐loaded injectable bioglass microsphere hydrogel technology to aid in the healing of bone abnormalities induced by periodontitis. Furthermore, quercetin may repair oxidative stress damage to OMSCs by modulating the mRNA changes of m6A‐modified Per1, regulating the oxidative stress milieu in periodontitis, and facilitating bone defect healing. This discovery paves the way for a therapeutic approach and mechanistic inquiry into the clinical treatment of periodontal alveolar bone defects.

## Experimental Section

See Supporting Information.

## Conflict of Interest

The authors declare no conflict of interest.

## Author Contributions

H.Z. and C.C. contributed equally to this work and are the first co‐authors. Y.Y. helped with sequencing data analysis. Y.Z., S.Y., Y.H., Y.Z., J.Z., and J.Z. helped with molecular experiments and animal experiments.

## Supporting information

Supporting Information

Supporting Information

Supporting Information

Supporting Information

Supplemental Movie 1

## Data Availability

The data that support the findings of this study are available from the corresponding author upon reasonable request.
