# Supplementary material for: Quercetin‐Loaded Bioglass Injectable Hydrogel Promotes m6A Alteration of Per1 to Alleviate Oxidative Stress for Periodontal Bone Defects
Source: Adv Sci (Weinh). 2024 May 15;11(29):2403412. doi: 10.1002/advs.202403412 (PMC11304245; doi:10.1002/advs.202403412)
Supplement: Supplementary file 1 — Supporting Information [file ADVS-11-2403412-s003.pdf]

ADVANCED SCIENCE
Open Access

Supporting Information

for *Adv. Sci.*, DOI 10.1002/advs.202403412

Quercetin-Loaded Bioglass Injectable Hydrogel Promotes m6A Alteration of Per1 to Alleviate Oxidative Stress for Periodontal Bone Defects

Huimin Zhu, Chao Cai, Yeke Yu, Yuning Zhou, Shiyuan Yang, Yue Hu, Yan Zhu, Jia Zhou, Jieyun Zhao, Hailong Ma, Yujie Chen* and Yuanjin Xu**

Supporting Information

Quercetin-loaded bioglass injectable hydrogel promotes m6A alteration of Per1 to alleviate oxidative stress for periodontal bone defects

Huimin Zhu[1, a], *Chao Cai*[1, b], *Yeke Yu*[c], *Yuning Zhou*[a], *Shiyuan Yang*[a], *Yue Hu*[a], *Yan Zhu*[a], *Jia Zhou*[a], *Jieyun Zhao*[a], *Hailong Ma*[*,c], *Yujie Chen*[*,d], *Yuanjin Xu*[*, a]

H. Zhu, Y. Zhou, S. Yang, Y. Hu, Y. Zhu, J. Zhou, J Zhao, Prof. Y. Xu.

[a] Department of Oral Surgery, Shanghai Ninth People's Hospital, Shanghai Jiao Tong University School of Medicine, College of Stomatology, Shanghai Jiao Tong University, National Center for Stomatology, National Clinical Research Center for Oral Diseases, Shanghai Key Laboratory of Stomatology, No. 639, Zhizaoju Rd, Shanghai 200011, China.

E-mail: drxuyuanjin@163.com

[b] Dr. C. Cai

Zhejiang Engineering Research Center for Tissue Repair Materials, Wenzhou Institute, University of Chinese Academy of Sciences, Wenzhou, Zhejiang 325000, China

[c] Y. Yu, H. Ma

Department of Oral Maxillofacial-Head and Neck Oncology, Shanghai Ninth People's Hospital, College of Stomatology, Shanghai Jiao Tong University School of Medicine, No 639, Zhizaoju Rd, Shanghai 200011, China.

E-mail: mahl21@sjtu.edu.cn

[d] Dr. Y. Chen

State Key Laboratory of Metal Matrix Composites, School of Materials Science and Engineering, Shanghai Jiao Tong University, Shanghai 200240, China.

E-mail: yujiechen@sjtu.edu.cn

[1] co-first author; [*] co-corresponding author.

1. Supplementary materials and methods

1.1 Reagents

MEM Alpha basic (Gibco, USA), Fetal Bovine Sera (Gibco, USA), 0.25% trypsin-EDTA (Gibco, USA), Cell Counting Kit-8 (Dojindo, Japanese), Calcein/PI Cell Activity and Cytotoxicity Assay Kit (Beyotime, China), Zoletil 50 (Virbac China), Lipofectamine™ 3000 (Invitrogen, USA), Opti-MEM (Gibco, USA), TRIzol™ Reagent (Invitrogen™, cat. no15596018), Anti-N6-methyladenosine (m6A) Antibody (Sigma-Aldrich, cat. no ABE572), Dynabeads Protein A (Invitrogen™, cat. no 10002D), Dynabeads Protein G (Invitrogen™, cat. no 10004D), EpiTM m6A immunoprecipitation kit (Epibiotek, cat. no R1804), Ethyl orthosilicate (TEOS) (Aladdin Reagent, China), Triethyl phosphate (TEP) (Aladdin Reagent, China), Cetyl trimethyl ammonium bromide (CTAB) (Aladdin Reagent, China), Triethanolamine (TEA) (Aladdin Reagent, China), Cyclohexane (Aladdin Reagent, China), Phosphate Buffer Solution (PBS) (Gibco, USA), Penicillin/Streptomycin Dual antibiotic (Gibco, USA), Dimethyl sulfoxide (Sigma, USA), Gelatin (type A bloom from porcine skin, Aladdin Reagent, China), Hyaluronic acid (Taitan, China), Carboxymethyl chitosan (Macklin, China), Methacrylic anhydride (Sigma, China), 2-hydroxy-2-methylpropiophenone (Aladdin Reagent, China). DPPH free radical detection kit (SAINT-BIO, China), Cell cycle and apoptosis analysis Kit (Yeasen, China).

1.2 Cell culture

Two-week-old SD rats were anesthetized and executed, soaked in 75% ethanol, and the mandibles were removed under aseptic conditions, and the muscle tissue on the surface of the mandibles was removed and rinsed twice with PBS solution. The mandibular ascending portion was excised, and the medullary

cavity was rinsed several times by aspirating a-MEM medium in a 1 mL syringe. The rinse solution was collected at 1000 rpm/min for a few min, and the supernatant was discarded and the cells were resuspended in PBS. The cells were inoculated with a-MEM containing 15% fetal bovine serum and penicillin at a cell density of $1*10^6$/mL in a culture dish and incubated at 37°C in a 5% CO_2 incubator. 24 h later, the cells were rinsed 2-3 times with PBS to remove unadhered cells. After that, the solution was changed once in 3 d. The cells were digested by 0.25% trypsin at 80% cell concentration and passaged, and the 2-6th generation cells were taken for subsequent experiments.

1.3 Multi-directional induction of stem cell in vitro

1.3.1 Osteogenesis induction

The uniformly inoculated rat OMSCs were incubated in an incubator at 37°C with 5% CO_2. When the cell fusion reached 70%-80%, remove the complete culture medium from the wells and add 2 mL of rat OMSCs osteogenic induction differentiation medium (Pricella, China) to the six-well plate. The osteogenic differentiation medium was changed every 3 d. After 4 weeks of induction, identification was performed.

1.3.2 Chondrogenesis induction

When the fusion of rat OMSCs reached 80-90%, the cells were counted by digestion with 0.25% trypsin and resuspended by adding chondrogenic differentiation-inducing complete medium. Aspirate 500 μL of cell suspension into a 15 mL centrifuge tube and centrifuge at 150 g for 5 min; after centrifugation without shaking or blowing the cell mass, carefully loosen the cap of the centrifuge tube to facilitate gas exchange. Incubate at 37°C in 5% CO_2. Change fresh chondrogenic differentiation induction solution

every 2-3 d and flick the cell mass after the fluid change to allow it to float off the wall. After 30 d of continuous induction, the chondrocytes can be fixed in neutral formaldehyde and sectioned by paraffin embedding.

1.3.3 Detection

For osteogenic differentiated cells, the cells were first washed twice with PBS, and each well was fixed with 2 mL of 4% paraformaldehyde solution for 30 min. Wash with PBS 2-3 times, followed by alizarin red staining. For chondrogenic differentiated cells, first fixed with neutral formaldehyde, paraffin-embedded sections were dewaxed to water, rinsed with distilled water and then stained with toluidine blue staining solution for 30 min, rinsed and terminated, and observed under the microscope.

1.4 Real-time polymerase chain reaction (RT-PCR)

Real-time PCR was performed as described in our previous study. Total RNA was purified from OMSCs by Trizol reagent. The cDNA was reverse transcribed from total RNA using the SMARTScribe™ reverse transcriptase System (TaKaRa) and was used for quantitative PCR amplification by Roche LightCycler® 480II PCR instrument (Basel, Switzerland). The primer sequences are shown in Table S1.

1.5 Western blotting

After lysing the proteins in RIPA with protease and phosphatase inhibitors, loading and DTT were added, and the samples were put in a metal bath at 95℃ for 9 min to fully denature the proteins. Protein electrophoresis was carried out using a premade gel (GenScript, China), and protein samples were chilled to room temperature before being sampled directly into the SDS-PAGE gel loaded wells.

Different electrophoresis settings were applied for each protein. When the bromophenol blue reached the bottom of the gel, the electrophoresis was stopped. For membrane transfer, PVDF membranes (Millipore) are used, and the specific transfer time is determined by the size of the protein. The membrane is immediately immersed in TBST (Tris-buffered saline containing 0.1% Tween-20) and washed for 1-2 min to remove the transfer solution from the membrane. Add a suitable quantity of QuickBlock™ western blocking solution (Beyotime, China), shake slowly for 10 min at room temperature. Refer to the primary antibody's instructions, dilute the primary antibody separately, incubate overnight at 4°C with moderate shaking, and wash for 5-10 min with TBST washing solution three times. Select GAPDH antibody as an internal reference. Secondary antibodies were added and incubated at room temperature for one hour. The ECL system (Thermo Fisher, USA) was used to visualize the final protein membranes. The following primary antibodies were used for western blot analysis: anti-OCN antibody (1:1000, Santa Cruz), Runx2 (1:1000, Santa Cruz), anti-Sox-9 (1:1000, Santa Cruz), anti- anti-Per1 (1:1000, Invitrogen), anti-GAPDH (1:3000, Cell Signaling Technology), anti-β-actin (1:3000, Cell Signaling Technology).

1.6 CCK-8 assay

Cell viability was performed as described previously. In brief, OMSCs cell viability was measured by the CCK-8 assay (Dojindo, Japan). Rat OMSCs were uniformly inoculated in 96-well plates, and after the cells reached the appropriate density, H_2O_2 stimulation was given for different times (no stimulation, 15 min, 30 min, 60 min). Subsequently, quercetin at different concentrations (0 μM, 2 μM, 4 μM, 8 μM) was given for 2 h of stimulation. Three replicates of each group were performed. 10 μL of CCK-8 solution was added to each well and incubated in a 37°C, 5% CO_2 incubator. Finally, the absorbance at 450 nm was measured by enzyme marker.

1.7 Flow assay of cell cycle

Cells were grouped as above, trypsin-digested cells were centrifuged for 5 min and collected. Cells were fixed with pre-cooled 75% alcohol 4°C, overnight. Remove alcohol and wash with PBS. Add 200ul PBS and 10ul RNAase then 200ul PI and incubate at 37°C for 30 minutes.

1.8 Flow assay of dead and live cells

Calcein AM-PI (Yeasen, 40747ES76, China) was used for apoptosis detection. Live cells were labeled with green fluorescence and dead cells were labeled with red fluorescence. Cells were inoculated in 24-well plates and divided into a negative control group (PBS), an oxidative stress-stimulated group (100 μM of H_2O_2 for 30 min), and a quercetin-treated group (100 μM of H_2O_2 for 30 min followed by 4 μM quercetin for 2 h). After the different treatments the intracellular medium was removed and washed twice. Add 100 μL of 2 μM Calcein AM and 4.5 μM PI working solution. incubate at 37°C for 15 min.

1.9 Reactive oxygen species (ROS) assay

Cells were inoculated in well plates and treated with 100 μM H_2O_2 for different times (0, 5, 10, 15, 30, 60 min). Subsequently, quercetin was used for 2 hours. ROS detection kit (Elabscience, China, item no.: E-BC-K138-F) was used to detect the intracellular ROS content. Prepare the appropriate reagents according to the instructions. Remove the cell culture medium, wash once with serum-free cell culture medium, add the appropriate volume of reagent 1 working solution, fully cover the cells, incubate the

cells for 30 min at 37°C and avoid light, remove the reagent 1 working solution, wash 2-3 times with serum-free cell culture medium to fully remove the ROS that have not entered the cells.

1.10 Reduced glutathione (GSH) assay

The Reduced Glutathione (GSH) Colorimetric Assay Kit was used for the assay. The procedure is approximately as follows: Prepare the appropriate liquid according to the instructions. Take 0.1 mL of the sample to be tested, add 0.1 mL of Reagent I, mix well, centrifuge at 4500 g for 10 min, take the supernatant for testing, and add 25 μL of Reagent III to the standard, assay and assay blank wells. Determination blank wells: add 100 μL of Reagent I; standard wells: add 100 μL of different concentrations of GSH standard solution; determination wells: add 100 μL of supernatant; add 100 μL of Reagent II to each well. The OD value was measured at 405 nm with an enzyme marker. Calculating the cellular GSH content according to the formula.

1.11 m6A RNA methylation quantification

Cells were divided into blank control, H_2O_2-stimulated and quercetin-treated groups, and subsequently assayed according to the kit instructions (EpiQuik™ m6A RNA Methylation Quantification Kit, USA), in the following steps: extract cellular RNA, fuse 200 ng of sample in DEPC water, add 80 μL of BS (Binding Solution) to each strip plate well, followed by 2 μL of NC (Negative Control) and 2 μL of PC (Positive Control). Add 80 ul of BS (Binding Solution) to each strip plate well, followed by 2 μL of NC (Negative Control) and 2 μL of PC (Positive Control), add 5 μL of sample, mix the reaction solution gently, seal the strip plate with Parafilm M and place it at 37 °C for 90 min. Remove the BS, add 150

μL of Diluted WB (Wash Buffer) and wash 3 times. Add 50 μL of Diluted CA, incubate for 1 h at room temperature, then remove Diluted Capture Antibody, wash 3 times with 150 μL of Diluted WB, add 50 μL of Diluted DA to each well, incubate for 30 min at room temperature. Remove Diluted Detection Antibody, 150 μL of Diluted WB washed 3 times, add 50 μL of Diluted Enhancer Solution. Incubate at room temperature for 30min, 150 μL of Diluted WB washed 5 times. Add 100 μL of Developer Solution. Add 100 μL of Stop Solution at room temperature and avoid light for 5 min and detect the OD value at 450 nm. The absolute quantification was calculated according to the formula.

1.12 m6A-RIP and m6A sequencing (MeRIP-seq) assay, RNA sequencing and data analysis

The assay was mainly carried out by SHBIO corporation (Shanghai, China). Total RNA was first extracted by Trizol, and after separation and purification, RNA interruption buffer was added, and the reaction was terminated by adding EDTA immediately after incubation at 70°C for 6 min. The fragmented RNA was purified and recovered using Zymo RNA clean and concentrator-5 kit, and the product was added to the buffer containing anti-N6-methyladenosine (m6A) antibody, protein A-magnetic beads, protein G-magnetic beads in precipitation buffer. The RNA obtained after immunoprecipitation, the products were subjected to ribosomal RNA removal, synthesis of first-strand cDNA by smart principle, PCR amplification to enrich library fragments, and DNA purification of magnetic bead library fragments to obtain ultra-microRNA methylation m6A detection libraries. The libraries were quality-checked using Bioptic Qsep100 Analyzer. The high-throughput sequencing platform of illumina NovaSeq 6000 with PE150 sequencing mode was used for sequencing. The filtered clean reads were compared with the reference genome of the corresponding species of the samples using HISAT2 software to obtain unique mapped reads for the next analysis. Enrichment region identification

analysis was performed using exomePeak software and IGV software. HOMER software was used to perform motif analysis of Peaks. For GO and KEGG analysis, we annotate the obtained m6A genes based on the database and calculate the significance level using Fisher's test to filter out the significant GO Term and the significant Pathway Term for m6A gene enrichment. differential Peak analysis was performed using differential methylation analysis with log2 (odds ratio), the log2 conversion value of fold change, was used to analyze locus changes. Where diff. log2. fc<0 is Hypomethylated, indicating that the site is demethylated under the treatment conditions, and diff. log2. fc>0 is Hypermethylated, indicating that the site is hypermethylated under the treatment conditions. For RNA-seq analysis, the gene expression values were normalized using FPKM (Fragments Per Kilo base Million Reads). Differential gene screening was performed using DESeq2 software with the screening conditions of |log2FC|>1 and FDR<0.05. Gene expression clustering analysis and GO and KEGG analysis were also performed as above.

1.13 Cell transfection

Per1 knockdown plasmids were constructed on OMSCs. Details of plasmid vector and mapping can be found in Table S1. OMSCs cells were transfected with Lipofectamine™ 3000 at 70-90% fusion rate and then changed to complete medium after 4 h for 48 h.

1.14 Construction of bioglass microspheres

In a round bottom flask, 54 mL of ionised water, 6 g of surfactant CTAB, and 0.18 mL of catalyst TEA were mixed well in a constant temperature water bath at 60°C. Following that, 6 mL of ethyl orthosilicate and 24 mL of organic solvent cyclohexane were combined and thoroughly agitated at room temperature.

The two liquids were progressively mixed before being placed in a constant temperature water bath at 60°C. The lower aqueous phase solution was slowly stirred. After 3 h of heating, 0.921 mL of triethyl phosphate was added and the reaction was continued. The top oil phase liquid was separated, and the bottom gel solution was centrifuged and washed with anhydrous ethanol and deionized water to produce a white gel precipitate that was freeze-dried to produce the precursor powder. To make the bioactive glass, a particular amount of precursor powder and calcium nitrate tetrahydrate were dissolved in anhydrous ethanol solution, shaken at 37°C for 24 h, then removed and heated at high temperature. Finally, transmission electron microscopy and scanning electron microscopy were used.

1.15 Synthesis of GelMA, HA-MA, and CMCS-MA

Type A gelatin from porcine skin was dissolved in PBS at 50°C to obtain a 10% w/v homogeneous solution. Methacrylic anhydride (MA) was then added to the gelatin solution at a rate of 0.5 mL/min, with continuous stirring, where 0.3 mL of MA was added per gram of gelatin. The mixture was continuously stirred for 4 h at 50°C. Finally, the solution was loaded into a dialysis bag (10–14 kDa) and dialyzed against deionized water for 3 d at 40°C to remove unreacted MA and any byproducts. The methacrylated gelatin (GelMA) solution was freeze-dried at -80°C and stored at a low temperature for further use. HA-MA and CMCS-MA were also synthesized according to the experimental protocol described above.

1.16 Synthesis of drug-loaded injectable hydrogel

1 g of bioglass was added to 40 μg/mL of quercetin solution, stirred overnight and centrifuged to collect the drug-loaded bioglass. Dissolve GelMA (2%), HA-MA (1%), drug-loaded bioglass (1%) and CMCS-

MA (1%) fully in PBS solution, then add UV initiator (0.2% 2-hydroxy-2-methylpropiophenone) and continue stirring until completely dissolved, then place the above solution under UV light (365 nm, 10 W power) irradiated for 20 min.

1.17 Loading and slow release of quercetin

The drug was mixed with bioglass in DMSO solution, sonicated for 20 min and stirred away from light for 24 h, after which the product was centrifuged and collected to obtain the drug loaded bioglass. The drug loaded bioglass was mixed and stirred well with the hydrogel precursor solution and placed under UV light for 10 min to obtain the composite hydrogel. A certain mass of composite hydrogel was weighed and loaded into a dialysis bag (3500 kDa cutoff) and then placed in 10 mL of saline, and 1 mL of the retardant solution was taken at different time points and the UV absorbance at 259 nm was measured. The concentration of the drug was converted from the standard curve.

1.18 Double staining of dead and live cells

Calcein-AM/PI double staining kit (Yeasen, 40747ES76, China) was used for the assay.OMSCs were incubated in advance with different concentrations of hydrogel (1 μL, 10 μL) in the immersion solution for different times (48 h, 72 h), and subsequently the cells were digested by trypsin, PBS wash for 3 times. 100 μL of staining working solution was added to each cell sample and incubated at 37°C for 30 min to observe green live cells and red dead cells, respectively.

1.19 DPPH free radical assay

Cells were divided into normal control group, H_2O_2 group (100 μM for 30 min), H_2O_2 + quercetin group (100 μM for 30 min, 4 μM quercetin for 2 h), H_2O_2 + i-BH group (100 μM for 30 min, 20 μL/100 μL i-BH for 72 h), H_2O_2 + i-BH-Q group (100 μM for 30 min, 20 μL/100 μL i-BH for 72 h). 0.8 mL of nitrogen radical extract was added per 5×10^6 cells, broken by ultrasound, centrifuged at 10000 rpm for 10 min, and the supernatant was taken for use. A standard working solution of 30 μg/mL of Vc with approximately 100% clearance was used as a positive control. For each group, 0.05 mL of cell supernatant was added to 0.45 mL of working solution, and the supernatant was allowed to stand at room temperature and protected from light for 30 min, and the absorbance values of each tube were detected at 500-530 nm by an enzyme counter.

1.20 Construction of periodontitis model

Six-week-old SD rats were used, and the cervical part of the maxillary second molar was ligated with 3-0 silk wire to create a periodontitis bone defect model. The injectable mesoporous microsphere sustained-release system was implanted into the bone defect area, and the rats were randomly assigned to one of three groups: a blank control group (periodontitis bone defect group), a simple material group (injectable mesoporous microsphere group), and a slow-release system group (quercetin/injectable mesoporous microsphere group). The rats were sacrificed after 4 weeks of feeding, and maxillary bone tissue was fixed for subsequent testing.

1.21 Micro CT detection

Maxillary bone scanning was performed with a Bruker SkyScan 1272, an instrument with fast scanning and easy control, enabling non-destructive 3D microscopic imaging of objects at an ideal resolution

condition. The experimental conditions were 70 kV voltage, 142 uA current, and scanning at a 20 μm scan fraction rate. The 3D image reconstruction was performed with N-Recon software, and 3D analysis was performed with CT-AN software.

1.22 H&E, Safranin solid green and TRAP stain

The bone tissue was fixed in 4% paraformaldehyde (PFA) solution for 24 h after the connective tissue was removed from the alveolar bone. The tissue was immersed in an EDTA decalcifying agent and changed with fresh solution on a daily basis, and decalcification was completed with a large-headed needle that could pierce the hard bone. By rinsing the tissue with running water for 24 h, the acidity was reduced. The tissue was then dehydrated and transparent with gradient ethanol and xylene before paraffin embedding, and cut into sections of 6 μm thickness for subsequent staining.

1.22.1 H&E staining

Dewaxed paraffin sections were washed, stained with hematoxylin for 3-8 min, then rinsed with tap water. After a few seconds of 1% hydrochloric acid alcohol fractionation, the sections were rinsed with tap water and restored to blue with 0.6% ammonia, rinsed with running water, and stained for 1-3 min in eosin staining solution. The parts were dehydrated before being sealed with neutral gum.

1.22.2 Safranin solid green staining

Paraffin tissue sections were deparaffinized to water, placed in solid green staining solution for 5-10 min, and washed to remove excess staining solution. Differentiation solution slightly soaked and washed slightly. The slices were put into saffron staining solution for 15-30 s, dehydrated quickly with anhydrous ethanol, transparent with xylene for 5 min, and sealed with neutral gum.

1.22.2 TRAP staining

Slices were dewaxed to water, drop-stained with Trap staining solution, and incubated at 37 °C for 20 min before being dehydrated, clear, and sealed with neutral gum.

1.23 Immunohistochemistry

Paraffin sections were deparaffinized to water, antigen repair solution was added, followed by incubation with 5% BSA (containing 0.2% Triton X-100) in a wet box for 30 minutes and rinsed three times on a shaker. The primary antibody was incubated overnight at 4 °C, and after rinsing, the secondary antibody was incubated at room temperature for 1 h protected from light, and finally visualized under an Olympus VS120 microscope. The information of antibodies was as follows: Mus-Nrf2 (Affinity, USA, BF8017, 1:100), Rab-Catalase (Sigma, USA, ZRB1771, 1:1000), Rab-Glut1 (CST, USA, 73015, 1:500), Mus-SOD2 (Proteintech, USA, 66474-1-1g, 1:5000), Rab-Per1 (Invitrogen, USA, PA5-119798, 1:100). alexa Fluor 488 and 594 IgG-HRP were used as secondary antibodies at a dilution of 1:1000.

1.24 Statistical analysis

All statistical analyses were conducted using GraphPad Prism 8.0 and SPSS v22.0 software. Two-tailed Student's t test was applied for comparisons between two groups, and one-way ANOVA with Tukey's multiple comparisons test was applied for multiple comparisons. Spearman's correlation analysis was used to perform the correlation analysis. All results were calculated in at least three independent experiments. All error bar values represent the standard deviation (SD) or standard error of mean (SEM). In all types of statistical analysis, p values < 0.05 were considered statistically significant

2. Supplementary Figures

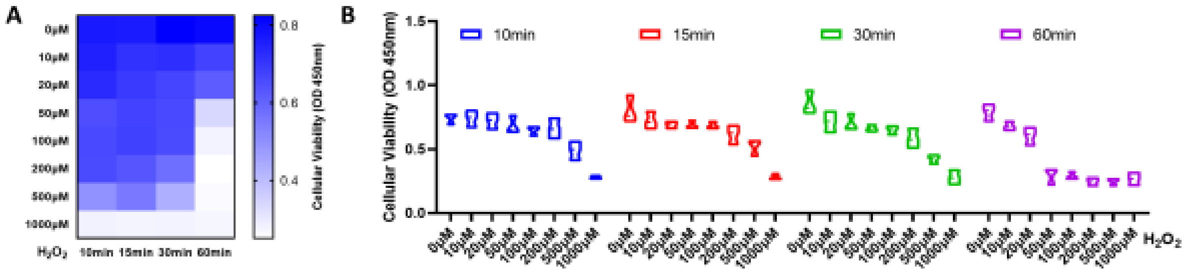

Figure S1. Heat map (A) and statistical analysis (B) of CCK-8 detection of OMSCs survival rate after different concentrations of H_2O_2 (10, 20, 50, 100, 200, 500, 1000 μM) acting for different periods (10, 15, 30, 60 min).

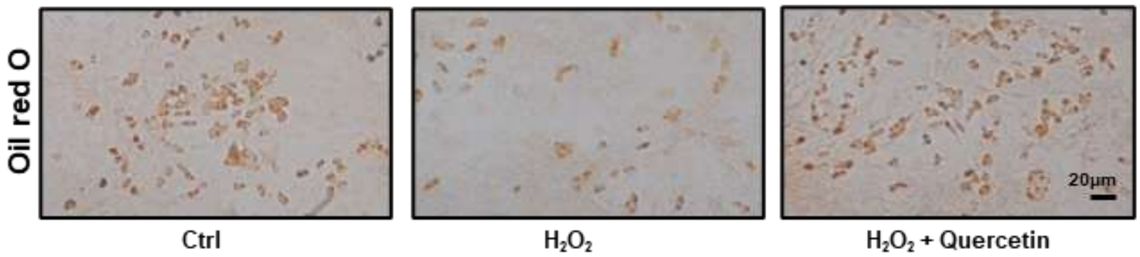

Figure S2. Oil red O staining of P2 generation OMSCs in the control group, oxidative stress group (100 μM H_2O_2 for 30 min, differentiation induction for 21 days) and quercetin treatment group (100 μM H_2O_2 for 30 min followed by 4 μM quercetin, differentiation induction for 21 days).

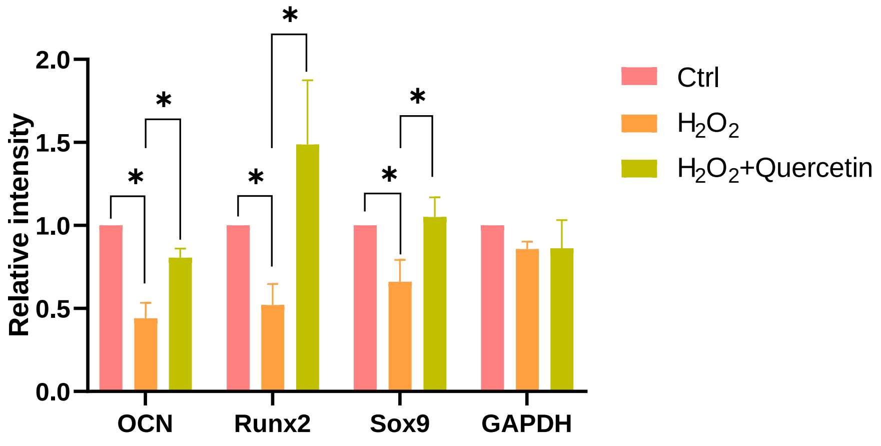

Figure S3. Statistical analysis of protein gray value area. *, p<0.05.

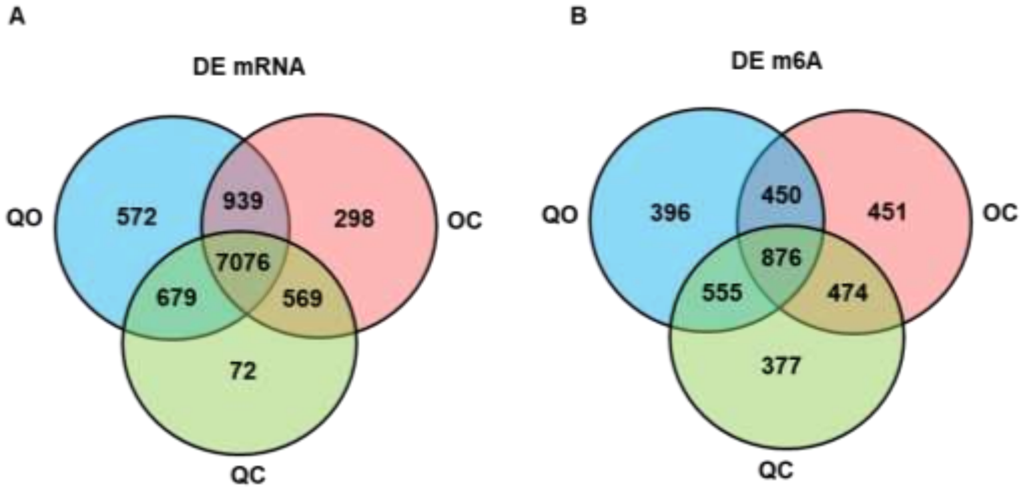

Figure S4. Two-by-two comparison of the number of differentially (DE) mRNA (A) and m6A modified (B) genes in the three groups Venn diagram. C, Control group; O, H_2O_2 treated group; Q, H_2O_2 and quercetin treated group.

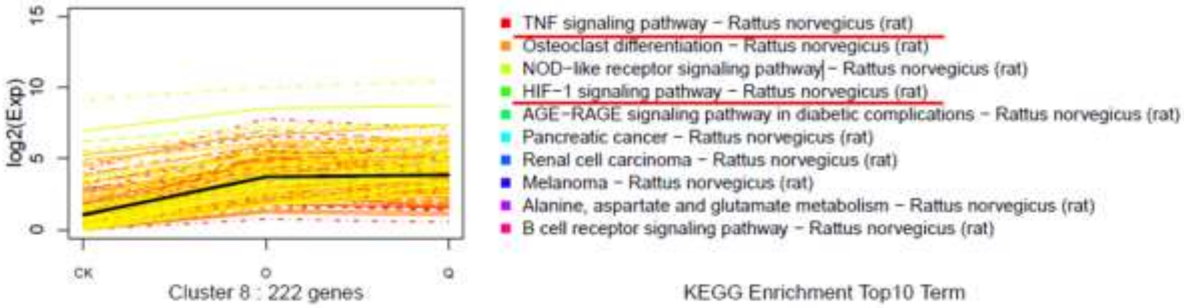

Figure S5. Kmeans clustering algorithm to analyze gene pathway enrichment in three groups with opposite mRNA changes.

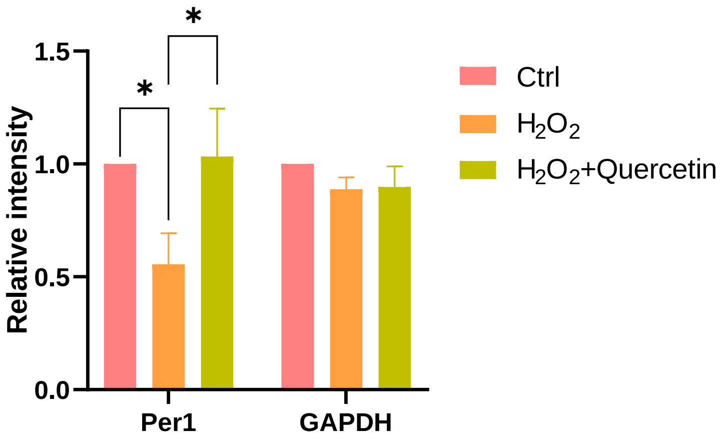

Figure S6. Statistical analysis of protein gray value area. *, $p<0.05$.

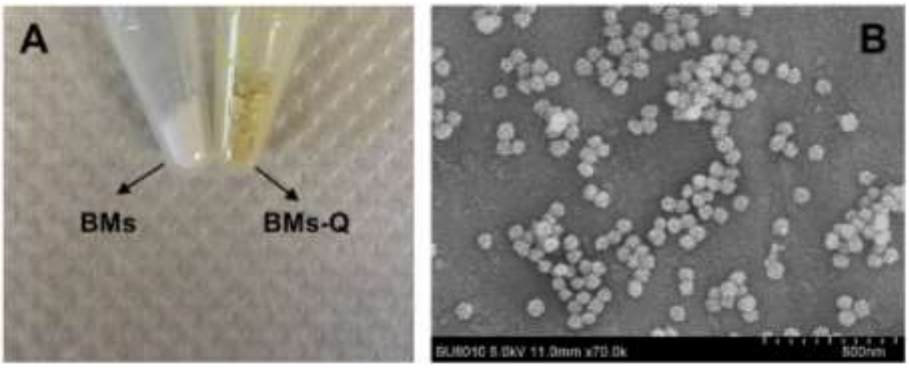

Figure S7. (A) The photos of pure bioglass and quercetin-loaded bioglass. (B) The SEM images of quercetin-loaded bioglass.

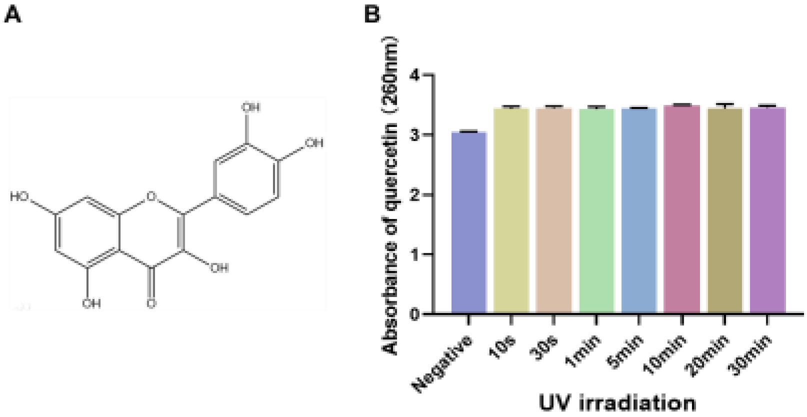

Figure S8. (A) Chemical structural formula of quercetin. (B) UV-vis absorption spectroscopy of quercetin solutions after different durations of UV illumination.

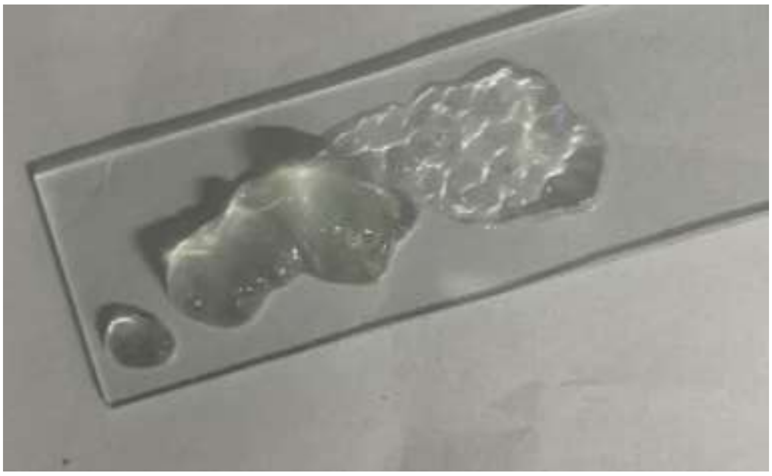

Figure S9. Picture of an injectable hydrogel formed after UV irradiation.

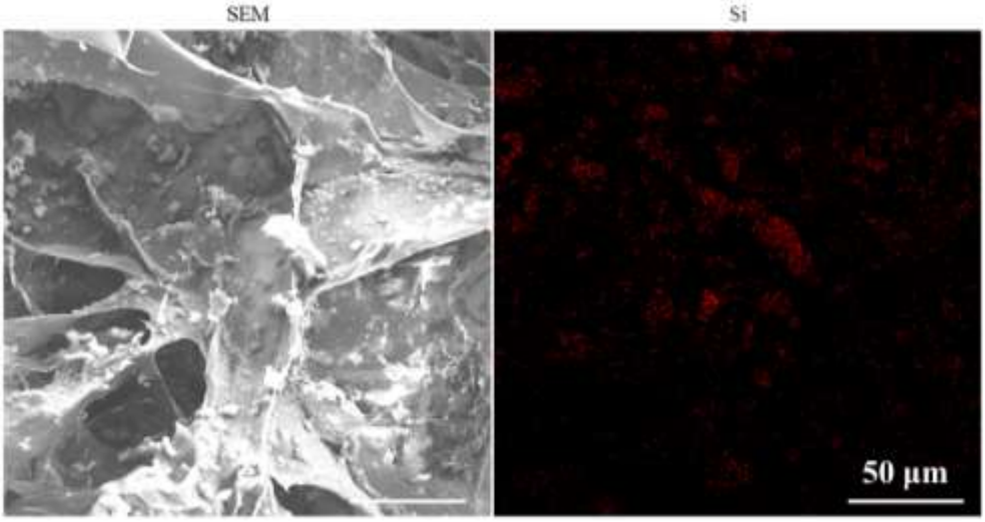

Figure S10. SEM and EDS images of the composite hydrogel.

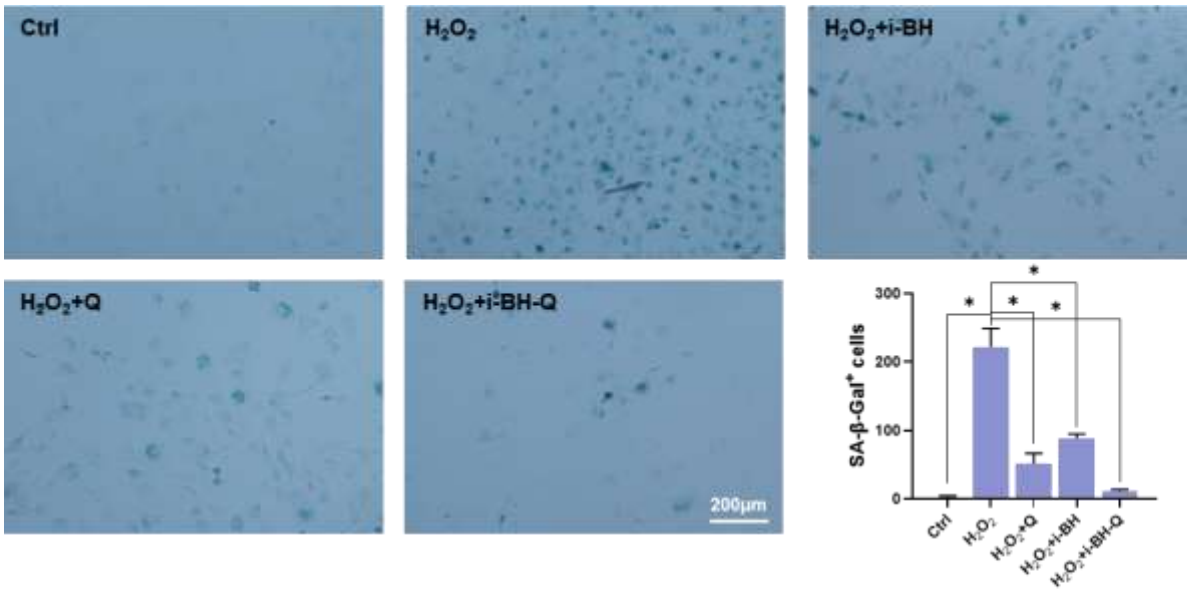

Figure S11. The number of SA-β-gal positive cells were detected in the control group, H_2O_2 group (100 µM action for 30 min), H_2O_2 + quercetin group (100 µM action for 30 min, 4 µM quercetin action for 2 h), H_2O_2 + i-BH group (100 µM action for 30 min, 20 µL/100 µL of i-BH action for 72 h), H_2O_2 + i-BH-Q group (100 µM action for 30 min, 20 µL/100 µL of i-BH-Q for 72 h). *, $p < 0.05$.
