# Supplementary material for: Quercetin‐Loaded Bioglass Injectable Hydrogel Promotes m6A Alteration of Per1 to Alleviate Oxidative Stress for Periodontal Bone Defects
Source: Adv Sci (Weinh). 2024 May 15;11(29):2403412. doi: 10.1002/advs.202403412 (PMC11304245; doi:10.1002/advs.202403412)

ADVANCED SCIENCE
Open Access

Supporting Information

for *Adv. Sci.*, DOI 10.1002/advs.202403412

Quercetin-Loaded Bioglass Injectable Hydrogel Promotes m6A Alteration of Per1 to Alleviate Oxidative Stress for Periodontal Bone Defects

Huimin Zhu, Chao Cai, Yeke Yu, Yuning Zhou, Shiyuan Yang, Yue Hu, Yan Zhu, Jia Zhou, Jieyun Zhao, Hailong Ma, Yujie Chen* and Yuanjin Xu**

Supplementary Table 1. Primers and sequences used in this study.

gene_name		Sequence (5' -> 3')
**Rat-ALP**	**Forward**	GGGGTCAAAGCCAACTACAA
	**Reverse**	CTTCCCTGCTTTCTTTGCAC
**Rat-RUNX2**	**Forward**	GCCGGGAATGATGAGAACTA
	**Reverse**	GGACCGTCCACTGTCACTTT
**Rat-OCN**	**Forward**	GTCCGCTAGCTCGTCACAAT
	**Reverse**	GAGGGCAGTAAGGTGGTGAA
**Rat-COL1**	**Forward**	GATGGTGCCAAAGGAGAT
	**Reverse**	AACAGCACCATCGTTACC
**Rat-SOX9**	**Forward**	GGCGGAGGAAGTCGGTGAAG
	**Reverse**	AGATGGCGTTAGGAGAGATGTGAG
**Rat-beta-actin**	**Forward**	CCCATCTATGAGGGTTACGC
	**Reverse**	TTTAATGTCACGCACGATTTC
**Rat-METTL3**	**Forward**	CGTAACCTATGCTGACCACTCCAAG
	**Reverse**	TGTGATAGTCCCTGCTGCCTCTC
**Rat-METTL14**	**Forward**	TTCGGGAGGGACAGCACTATCAG
	**Reverse**	GCCTCTCAATTTCCTCGGTACATCC
**Rat-WTAP**	**Forward**	GAGAACATCCTTGTCATGCGGCTAG
	**Reverse**	TCGGCTGCTGAACTTGCTTGAG
**Rat-RBM15**	**Forward**	CCGTCACTGCTTATGAGCCACTG
	**Reverse**	CAAGATGCCGTCCTCCACTTTCC
**Rat-YTHDC1**	**Forward**	CACACAAGCCGTTGTCAGTG
	**Reverse**	CGATCACGACCTCTGTCTCG
**Rat- YTHDC2**	**Forward**	AGTAGGGCAACAGACACAGC
	**Reverse**	AGTAGATCCAGAACCCGCCT
**Rat- YTHDF1**	**Forward**	GCCAGGAGGAAGAGGAGGTAGTG
	**Reverse**	CGGAGACAGCACCAAGCATACAG
**Rat- YTHDF2**	**Forward**	TTGCCTCCACCTCCACCACAG
	**Reverse**	CCCATTATGACCGAACCCACTGC
**Rat- YTHDF3**	**Forward**	CTCTCGGGTTACGGTGAAGAATGTC
	**Reverse**	ATGTTGGCTGTTAGGTGCGATGG
**Rat-FLT1**	**Forward**	ATGCGCATGAGAACACCAGA
	**Reverse**	CACAAGTTCAGCAAACCGGG
**Rat-GSPT1**	**Forward**	AAAACAGTAGAGGTGGGCCG
	**Reverse**	ACCAGTACAGCCAAGTCAGC
**Rat-IRAK1**	**Forward**	CTTTCCTGGCCTCAACGACT
	**Reverse**	TACTGCTCTGGCTTGGGTTG
**Rat-PER1**	**Forward**	AACAGCCACGGTTCTCAGAG
	**Reverse**	CAGCCCCAATCCATCCAGTT
**Rat-TRIO**	**Forward**	AGCAGTTCCAGCACGCTATT
	**Reverse**	ACCTTCTCTGCACAGTCACG
**Rat-LGSF3**	**Forward**	GGATGGAGAGTGGCAGATCG
	**Reverse**	TGTCCGGGAGATAGCTGTGA

shRNA vector mapping and target sequence for pLV3-U6-Per1（rat）-shRNA-CopGFP-Puro.

gagagcagcaagagtacaaacTTCAAGAGAgtttgtactcttgctgctctc

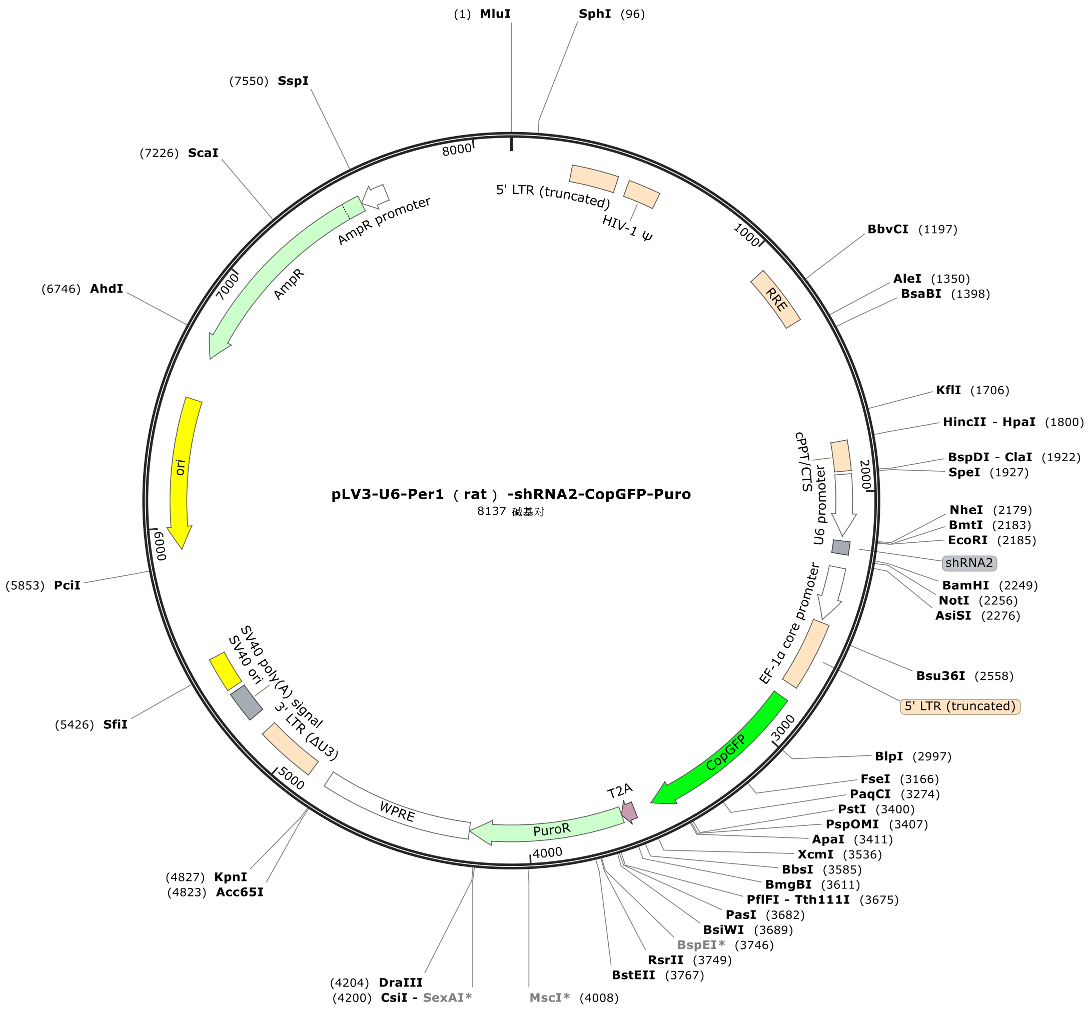

Supplement: Supplementary file 2 — Supporting Information [file ADVS-11-2403412-s001.pdf]
